# An application of upscaled optimal foraging theory using hidden Markov modelling: year-round behavioural variation in a large arctic herbivore

**DOI:** 10.1186/s40462-020-00213-x

**Published:** 2020-06-05

**Authors:** Larissa T. Beumer, Jennifer Pohle, Niels M. Schmidt, Marianna Chimienti, Jean-Pierre Desforges, Lars H. Hansen, Roland Langrock, Stine Højlund Pedersen, Mikkel Stelvig, Floris M. van Beest

**Affiliations:** 1 Department of Bioscience, Aarhus University, 4000 Roskilde, Denmark; 2 Arctic Research Centre, Aarhus University, 8000 Aarhus, Denmark; 3 Department of Business Administration and Economics, Bielefeld University, 33615 Bielefeld, Germany; 4 Natural Resource Sciences, McGill University, Ste Anne de Bellevue, Quebec, H9X 3V9 Canada; 5 Cooperative Institute for Research in the Atmosphere, Colorado State University, Fort Collins, CO 80523 USA; 6 Department of Biological Sciences, University of Alaska Anchorage, Anchorage, AK 99508 USA; 7 Copenhagen Zoo, 2000 Frederiksberg, Denmark

**Keywords:** Hidden Markov modelling, Behavioural state classification, Seasonality, Activity budgets, Arctic ungulate, Optimal foraging theory

## Abstract

**Background:**

In highly seasonal environments, animals face critical decisions regarding time allocation, diet optimisation, and habitat use. In the Arctic, the short summers are crucial for replenishing body reserves, while low food availability and increased energetic demands characterise the long winters (9–10 months). Under such extreme seasonal variability, even small deviations from optimal time allocation can markedly impact individuals’ condition, reproductive success and survival. We investigated which environmental conditions influenced daily, seasonal, and interannual variation in time allocation in high-arctic muskoxen (*Ovibos moschatus*) and evaluated whether results support qualitative predictions derived from upscaled optimal foraging theory.

**Methods:**

Using hidden Markov models (HMMs), we inferred behavioural states (foraging, resting, relocating) from hourly positions of GPS-collared females tracked in northeast Greenland (28 muskox-years). To relate behavioural variation to environmental conditions, we considered a wide range of spatially and/or temporally explicit covariates in the HMMs.

**Results:**

While we found little interannual variation, daily and seasonal time allocation varied markedly. Scheduling of daily activities was distinct throughout the year except for the period of continuous daylight. During summer, muskoxen spent about 69% of time foraging and 19% resting, without environmental constraints on foraging activity. During winter, time spent foraging decreased to 45%, whereas about 43% of time was spent resting, mediated by longer resting bouts than during summer.

**Conclusions:**

Our results clearly indicate that female muskoxen follow an energy intake maximisation strategy during the arctic summer. During winter, our results were not easily reconcilable with just one dominant foraging strategy. The overall reduction in activity likely reflects higher time requirements for rumination in response to the reduction of forage quality (supporting an energy intake maximisation strategy). However, deep snow and low temperatures were apparent constraints to winter foraging, hence also suggesting attempts to conserve energy (net energy maximisation strategy). Our approach provides new insights into the year-round behavioural strategies of the largest Arctic herbivore and outlines a practical example of how to approximate qualitative predictions of upscaled optimal foraging theory using multi-year GPS tracking data.

## Background

To improve reproductive success and survival, wild animals adjust their behaviour and scheduling of activities to varying resource availability, environmental conditions and risk levels [1]. Assessing how animals fine-tune their behaviour to fluctuating conditions is thus fundamental to our understanding of how species cope with ecological constraints. This is especially urgent in the context of climate change, which may alter the range of biotic and abiotic conditions animals encounter. Arctic and alpine herbivores are challenged by some of the greatest seasonal differences in foraging, light and climatic conditions. Consequently, movement behaviour and foraging strategies are expected to vary markedly according to daily, seasonal, and interannual changes in local conditions [2–5].

Balancing trade-offs between energetic costs and gains, predation risk and environmental constraints, animals are constantly faced with decisions regarding optimal foraging timing, space and diet [6]. Traditionally, optimal foraging theory (OFT) distinguished between energy intake maximisation and time minimising strategies [6, 7]. Energy-maximising animals optimise energy intake by allocating most of their available time to feeding, whereas time-minimisers should only feed for the time necessary to satisfy minimum energetic requirements. While the former strategy yields the greatest amount of energy for maintenance, growth and reproduction, the latter provides the minimum amount of energy required to fulfil basic energetic needs while allowing higher time allocation to behaviours that improve survival, such as staying inactive during certain periods of the day to reduce the risk of predation. For herbivores in extreme environments, constraints such as adverse weather conditions may also be energetically limiting factors, and a net energy maximisation (i.e. energy conservation) strategy has therefore been proposed as additional potential optimal foraging strategy [2, 5, 8, 9]. This strategy is identical to the energy intake maximisation strategy as long as foraging provides a net energy gain. However, when the gained benefits do not outweigh the energetic costs of the foraging effort, animals should attempt to conserve energy instead of maximising intake. As constraints on foraging decisions can vary considerably between seasons, animals may adopt different strategies over the course of the year [5].

Conventionally, studies testing predictions of OFT focus on fine-scale foraging behaviour, usually assessed in experimental settings with direct observational data collected over relatively short periods and at small spatial scales. However, foraging behaviour of free-ranging animals follows a hierarchy of spatial and temporal scales, from bites at the food patch level to larger movements, representing foraging decision-making at daily, seasonal, and annual scales [10, 11]. Assessing optimality based on the maximisation of short-term gains may be misleading when considering longer time scales, and Owen-Smith et al. [12] therefore proposed an ‘upscaled’ approach to OFT, where the basic principles of OFT also apply to larger-scale movement behaviour. This approach is especially applicable to terrestrial herbivores, as vegetation usually remains constant in its spatial distribution, but may vary substantially in both availability and quality over time [12]. Indeed, interpreting activity budgets and behavioural responses to environmental conditions against qualitative predictions derived from (upscaled) OFT has proven useful to approximate seasonal behavioural strategies and to identify key constraints on foraging [2, 8, 13].

Thus far, studying the behaviours of wild animals and drivers thereof has been difficult, especially in remote regions and over time periods covering seasonal as well as interannual variability in environmental conditions. However, improvements in tracking technologies now allow recordings of high-precision animal movements over extended periods, independent of weather and light conditions even in inaccessible regions, while greatly reducing sampling or observer bias [14]. Simultaneously with these technological advancements, behaviour-focused modelling approaches have evolved, designed to detect different behaviours from telemetry data and to investigate their relationship with environmental conditions [15]. In particular, hidden Markov models (HMMs) have emerged as flexible behaviour-based tools for the analysis of regular observational time series driven by underlying, serially correlated states [16]. Moreover, physics-based numerical models explicit in space and through time (e.g. MicroMet and SnowModel [17, 18]) are increasingly capable of providing realistic environmental data at resolutions and extents relevant for ecological applications [19, 20], i.e. data traditionally unavailable from in situ measurements. Combined, these developments in data acquisition and analyses are greatly improving our ability to assess wildlife-environment interactions.

The muskox (*Ovibos moschatus*) is the largest arctic herbivore, well adapted to a cold and highly seasonal environment [21]. Due to the remoteness of its habitats, and the challenging environmental conditions and long periods of polar night throughout most of its range, detailed behavioural and matching environmental data covering several years with complete seasonal cycles were thus far lacking. In this study, we take advantage of a unique data set of multi-year movements of 19 female muskoxen tracked with GPS (Global Positioning System) collars in northeast Greenland (28 muskox-years, with 153–1062 observation days/animal) and apply HMMs to infer likely behavioural states (resting, foraging, relocating) from step lengths and turning angles between hourly positions. Our objectives were to (a) quantify diel, seasonal, and interannual variation in activity budgets and to (b) investigate how behavioural time allocation and state-switching are influenced by environmental conditions, using an extensive set of spatially explicit and/or temporally dynamic environmental variables. Finally, we also aimed to (c) evaluate whether the emerging behavioural patterns follow qualitative predictions derived from upscaled OFT. To do so, we formulated season-specific predictions for each of the foraging strategies proposed by OFT (see Table 1) against which we compared our results.

**Table 1:** Predictions for expected patterns in time allocation, state occupancy probabilities and activity scheduling if muskoxen were to follow either of the three proposed strategies according to optimal foraging theory, for the summer and winter season, respectively

Summer season (snow-free)	Winter season (snow-covered)
**Energy intake maximisation strategy**: muskoxen aim to maximise energy intake (i.e. time spent foraging and forage quality), only limited by digestive physiological constraints (i.e. time required for rumination)	
S1_INTAKE_: time allocation only influenced by forage quality/quantity (e.g. landcover, NDVI) since forage quality/quantity determines time required for rumen fill and rumination	W1_INTAKE_: time allocation only influenced by forage quality/quantity/accessibility (e.g. landcover, snow depth) since forage quality/quantity/accessibility determines time required for rumen fill and rumination
S2_INTAKE_: probability of foraging remains constant independent of changes in environmental conditions (e.g. temperature, wind)	W2_INTAKE_: probability of foraging/resting remains constant independent of changes in environmental conditions (e.g. temperature, snow depth)
S3 _INTAKE_: no specific daily scheduling of activities	W3_INTAKE_: no specific daily scheduling of activities
S4_INTAKE_: no interannual differences in time allocation	W4_INTAKE_: no interannual differences in time allocation
**Time minimisation strategy**: muskoxen only forage the minimum required time to satisfy basic energetic needs, while reducing e.g. risk of predation	
S1_TIME_: time allocation/state switching mainly influenced by forage quality/quantity (e.g. landcover, NDVI), time of day and light conditions	W1_TIME_: time allocation/state switching mainly influenced by forage quality/quantity/accessibility (e.g. landcover, snow depth), time of day and light conditions
S2_TIME_: proportion of time spent foraging decreases with increasing forage quality/quantity as same foraging effort yields higher energetic gains	W2_TIME_: proportion of time spent foraging increases with decreasing forage quality/quantity/accessibility to compensate for reduced energetic gains of foraging effort
S3_TIME_: specific daily scheduling of activities indicates avoidance of periods with e.g. higher risk of predation	W3_TIME_: specific daily scheduling of activities indicates avoidance of periods with e.g. higher risk of predation
**Net energy maximisation strategy**: muskoxen aim to maximise energy intake but switch to resting (i.e. energy conservation) as soon as constraints/costs of foraging outweigh gains of foraging effort	
S1_NET_: time allocation/state switching mainly influenced by forage quality/quantity and environmental conditions representing constraints	W1_NET_: time allocation/state switching mainly influenced by forage quality/quantity/accessibility and environmental conditions representing constraints
S2_NET_: probability of foraging decreases with environmental conditions causing thermal stress or insect harassment (e.g. high temperature, low wind speed)	W2_NET_: probability of resting increases with conditions causing heat loss (e.g. low temperature, high wind speed) or increasing energetic costs of movement and forage access (e.g. deep snow)
S3_NET_: specific daily scheduling of activities indicates avoidance of daily periods during which constraints peak (e.g. highest temperatures)	W3_NET_: less pronounced specific daily scheduling of activities because peaks in constraints (e.g. temperature/snow depth) do not necessarily follow regular daily patterns
S4_NET_: interannual differences in time allocation depending on interannual differences in the strength of environmental constraints	W4_NET_: interannual differences in time allocation depending on interannual differences in the strength of environmental constraints

## Methods

### Study area and study species

**Fig. 1:**
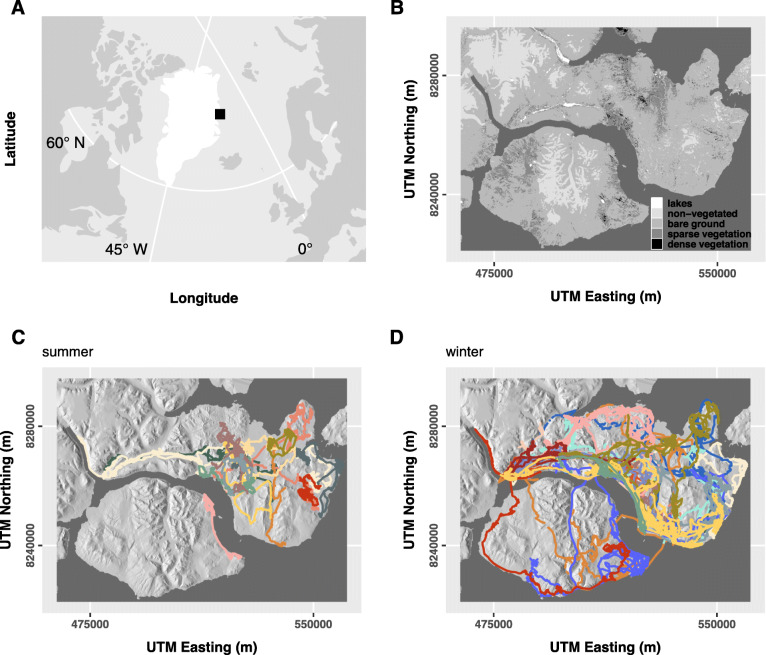
**a** Map indicating the study area (black rectangle) in northeast Greenland (white). **b** Map of the study area in detail (WGS 84, UTM zone 27), showing the distribution of landcover types (note that in the statistical models, lakes, non-vegetated and bare ground were pooled to ‘bare ground’). For the distribution of remaining static covariates, see Additional file 1: Fig. S1. **c** Muskox tracks during the snow-free summer and **d** snow-covered winter period across years, colour-coded by animal ID (within season). For an overview of muskox observations per season and year, see Additional file 1: Figs. S2-S3

The study area in northeast Greenland (approx. 5000 km^2^; Fig. 1 a-b) is characterised by a high-arctic climate with pronounced variability in light and weather conditions throughout the year. The mean annual ambient temperature is − 9 °C (1997–2017), peaking in July (monthly mean of 6.6 °C) and dropping to lowest values in February/March (− 20 °C). Snow typically covers the ground from early September to early June, but interannual variation in snow accumulation and duration of snow cover is considerable [22]. Broad valleys separated by fjords and mountains up to 1600 m in elevation determine the landscape’s topography. The vegetation consists of different tundra habitat types of varying productivity [23]. Muskoxen are the only large herbivores in the study area. Considered sedentary [24], the resident population was estimated to range between 2900 and 4600 individuals in 1990 [25], with mean annual densities varying between 1 and 3 individuals km^− 2^ between 1996 and 2013 [26]. In summer, muskoxen mainly consume energy-rich graminoids [27, 28], whereas shrubs, but also graminoids, are important in winter [29, 30]. Winter diets may vary substantially between years depending on snow conditions [28], but also over the course of the winter, with lower diet quality in late as compared to early winter [30].

### Collection of muskox movement data

In October of 2013 and 2015, *n* = 14 and *n* = 5 adult female muskoxen were fitted with GPS collars (Tellus Large; Followit Lindesberg AB, Sweden), respectively (for a detailed description of collaring procedures, see [24, 31]). Note that a total of 15 females were collared in 2015, but 10 of the collars, fitted with a GPS unit from a different manufacturing batch, exhibited lower positional accuracy (location error of 19.6 m (SD = 25.4) as opposed to 8.04 m (SD = 7.23)) and were therefore excluded from analyses to minimise misclassification of behavioural states. Collars were programmed to record one position per hour.

### Processing of movement data

Hourly location data were screened for impossible movements (detailed in [24]). Collar fix rate was generally high (98.5%), but in some instances, GPS fixes could not be obtained for several consecutive hours. If observation gaps exceeded 10 h, we split the movement tracks (*n* = 19) into bursts (*n* = 32) to keep time-series regularization (required for HMMs) and consequently needed interpolation of environmental covariate values for missing observations to a minimum. To account for potential snow-related differences in movement characteristics (e.g. shorter step length when moving in snow), bursts were subsequently divided into ‘seasonal’ bursts on the individual level: bursts were characterised as ‘snow-free’ (hereafter referred to as ‘summer’) for the period between the first and last 48 h where an individual did not encounter snow, and otherwise as ‘snow-covered’ (‘winter’) (see environmental data section on how snow conditions were acquired). Indeed, mean step lengths were over 80% longer in summer vs. winter (155.2 m v. 85.7 m, respectively). All seasonal bursts with less than 4 full weeks of consecutive observations were excluded from analyses (*n* = 7). Combined, 13.03% of observations were removed from the initial GPS dataset, and a total of 242,378 observations (n_summer_ = 28,165, n_winter_ = 214,213) divided into 70 seasonal bursts (Additional file 1: Fig. S2) were included in the final analyses, corresponding to 28 muskox-years with 153–1062 observation days/animal. For an overview of observations per season and animal ID, see Fig. 1 C-D and Additional file 1: Fig. S3/Table S1.

### Environmental data

To assess how muskox time allocation and state-switching probabilities were influenced by environmental conditions, we considered a wide range of spatially explicit and/or temporally dynamic environmental covariates known to influence ungulate behaviour (e.g. [4, 32]), detailed in Table 2. The temporally dynamic and spatially explicit meteorological covariates (air temperature, snow depth, wind speed, wind direction, total precipitation, see Table 2) were modelled for the study area and period using SnowModel and MicroMet (see [34] for details). Values for all covariates were extracted based on muskox GPS positions, and associated timestamps for temporally dynamic covariates. While muskox locations were recorded hourly, temporally dynamic covariates were available at a 3-hourly or daily resolution, respectively. However, assuming that meteorological conditions do not change substantially within 3 h and given that muskoxen might change position in space and thereby encounter different covariate values (i.e. spatial variation), we decided not to resample the muskox location data. Snow depth was only included in the analyses of winter bursts, whereas NDVI was only available in the absence of snow and hence solely included as covariate in the analyses of summer bursts. As NDVI reflected temporal dynamics in NDVI over the course of the summer season, we included it in addition to the temporally static NDVI-based landcover classification (see Table 2) to investigate if/how behavioural variation in muskoxen was related to seasonal changes in vegetation greenness. Any missing covariate values were filled in by linear interpolation. Considered covariates did not exhibit collinearity issues (i.e. Pearson correlation coefficient < |0.6|).

**Table 2:** Overview of covariates considered in the HMMs for the snow-free summer and snow-covered winter bursts

Covariate type	Covariate	Description	Biological effect	Data type	Spatial/temporal resolution	Data source
**temporal**	time of day	hour of the day	diel variation in environmental conditions, associated with predation risk levels	continuous	hourly	
	Julian day	day of the year	proxy for fine-scale seasonal variation in environmental conditions and diet quality	continuous	daily	
	year	season-year (e.g. winter season 2013/2014, summer season 2014)	interannual variation in environmental conditions	categorical	annual	
	light	light conditions (daylight or darkness) at time of observation	light, visibility, associated with predation risk levels	categorical	hourly	determined using ‘streamMetabolism’ package in R
**static**	landcover type	NDVI-derived landcover classification (NDVI ≥0.35 = ‘dense vegetation’, 0.1–0.35 = ‘sparse vegetation’, < 0.1 = ‘bare ground’ (including non-vegetated areas such as glaciers, perennial snow and lakes))	associated with plant productivity, forage abundance	categorical	30 m	vegetation classes classified based on NDVI, using Landsat 4-5TM satellite image, dated 17 July 2009; non-vegetated derived from 1:100.000 topographic maps, field measurements from study area [23, 33]
	elevation (m.a.s.l.)	elevation above sea level	associated with plant productivity and snow accumulation	continuous	30 m	ASTER Global Digital Elevation Model (DEM) Version 2 (https://asterweb.jpl.nasa.gov/gdem.asp)
	terrain ruggedness (index)	mean of the absolute differences between the value of a cell and the value of its 8 surrounding cells, i.e. measure of terrain heterogeneity	associated with vegetation heterogeneity and variation in snow conditions	continuous	30 m	calculated from DEM using ‘terrain’ function in ‘raster’ package in R
	distance to coast (m)	Euclidian distance to coastline	proxy for coast-inland gradients in e.g. precipitation, temperature	continuous	30 m	calculated from DEM using ‘raster’ package in R
	hillshade (unitless)	amount of incoming radiation, combining slope and aspect	associated with local temperature, plant productivity and snow melt dynamics	continuous	30 m	calculated from DEM using ‘hillShade’ function in ‘raster’ package in R
**dynamic**	snow depth (m)	snow depth	associated with forage accessibility and costs of foraging/movement	continuous, modelled	300 m, 3 h	MicroMet high-resolution meteorological model coupled with SnowModel snow-evolution modelling tool [17, 18, 34]
	ambient temperature (°C)	ambient air temperature (2 m above ground surface)	thermal conditions, associated with insect harassment			
	wind speed (m/s)	wind speed (2 m above ground surface)	associated with thermal conditions (windchill effect) and insect harassment			
	wind direction (degrees from north)	wind direction (2 m above ground surface)	associated with thermal conditions (windchill effect)			
	precipitation (mm)	precipitation (rainfall or snow) at time t	precipitation, associated with thermal conditions			
	NDVI (index)	Normalized Difference Vegetation Index (NDVI)	measure of vegetation greenness, related to vegetation growth and aboveground biomass [35]	continuous, observed	300 m, daily	Moderate Resolution Imaging Spectroradiometer (MODIS) Daily Surface Reflectance [34]

### Statistical analyses

To analyse muskox movement patterns over time, we fitted separate bivariate HMMs to the hourly-observed step lengths and turning angles for the summer and winter bursts, respectively. HMMs assume the observed movement patterns to be driven by an underlying latent state sequence (i.e. a finite-state Markov chain). These data-driven states can be interpreted as proxies for the animals’ unobserved behavioural modes [16]. Step lengths and turning angles were modelled using gamma and von Mises distributions, respectively, in each case conditional on the underlying state. As neither observed movement variables (Additional file 1: Fig. S4) nor initially explored modelled state-dependent distributions (Additional file 1: Fig. S5) revealed large differences between individuals’ movement patterns, we did not explicitly account for individual variation between animals in the HMMs. To assess potential spatio-temporal association between individuals (i.e. joint movements), we calculated the percentage of simultaneous positions where two animals were less than 100 m apart (group definition provided in [26]), using the global proximity analysis as implemented in the wildlifeDI R package. On average, 0.68% [range: 0–22%] of simultaneous summer and 0.67% [0–24%] of simultaneous winter GPS fixes were located within the defined distance threshold, and we therefore considered individuals to move independently of each other (see also Additional file 1: Fig. S2 [24];).

We used the HMMs to detect and classify the three most commonly observed major behavioural states within the daily activity patterns of muskoxen and other ungulate species (e.g. [32, 36]), namely resting (state 1), foraging (state 2) and relocating (state 3). We also explored HMMs with two, four and five states (Additional file 1: Figs. S6-S7), but although the Bayesian Information Criterion (BIC) favoured the 5-state model, we found that the HMMs with three states were the most reliable to interpret in a biologically meaningful way while still providing good model fit (Additional file 1: Fig. S9; for a more detailed discussion of state-selection in HMMs, see [37]). To investigate the influence of environmental conditions on muskox movement behaviour, the state transition probabilities were expressed as functions of the covariates using a multinomial logit link function with categories representing the different states the process might switch to [15]. Forward selection based on BIC was used to determine the influence of 14 covariates considered in each of the seasonal HMMs. To capture their periodic nature, we included sine and cosine terms for cyclic covariates (e.g. time of day). All HMMs were fitted in R (version 3.6.0 [38]) via numerical likelihood maximisation using the moveHMM package [39]. To avoid local maxima, we fitted each model with 30 sets of random starting values and chose the one with the highest log-likelihood value in each case.

Based on the final seasonal models, we decoded the latent states using the Viterbi algorithm [40], which provides the most likely state sequence given the model and thus the basis for calculating activity budgets and duration of behavioural bouts. Furthermore, for each of the covariates, we calculated the stationary probabilities of state occupancy as a function of the covariate values [41], with the other continuous covariates held fixed at their respective seasonal means, and the categorical covariates set to a reference category.

### Approximation of foraging strategies

To evaluate whether the observed patterns in time allocation, state occupancy probabilities and activity scheduling followed either of the three foraging strategies (energy intake maximisation, time minimisation, net energy maximisation) proposed by OFT, we formulated qualitative season-specific predictions (Table 1) against which we compared our results (Table 3).

**Table 3:** Summary of how results support predictions (Table 1) for expected patterns in time allocation, state occupancy probabilities and activity scheduling if muskoxen were to follow either of the three proposed strategies according to optimal foraging theory, for the summer and winter season, respectively

summer season (snow-free)			winter season (snow-covered)		
prediction	supported	reasons for support or rejection	prediction	supported	reasons for support or rejection
S1_INTAKE_	partially	- time allocation strongly (but not only) influenced by foraging conditions (landcover, ruggedness) - short resting bout duration	W1_INTAKE_	partially	- time allocation influenced by forage conditions (landcover, ruggedness) - long resting bout duration
S2_INTAKE_	yes	- no covariates selected that represent potentially constraining environmental conditions (e.g. temperature)	W2_INTAKE_	no	- time allocation not independent of potentially constraining environmental conditions (snow, temperature, wind speed)
S3 _INTAKE_	yes	- no specific daily scheduling of activities	W3_INTAKE_	no	- distinct daily scheduling of activities
S4_INTAKE_	yes	- year not selected as covariate	W4_INTAKE_	partially	- year selected as covariate - no pronounced interannual variation in activity budgets
S1_TIME_	partially	- light and foraging conditions (landcover, ruggedness) strongly influence time allocation - time of day not selected as covariate	W1_TIME_	partially	- time allocation influenced by time of day, forage (landcover, ruggedness) and light conditions
S2_TIME_	no	- time spent foraging is constantly high throughout summer	W2_TIME_	no	- foraging activity decreased over course of the winter (i.e. with declining forage quality, see Schmidt et al. 2018)
S3_TIME_	no	- no specific daily scheduling of activities during midnight sun period	W3_TIME_	yes	- distinct daily scheduling of activities
S1_NET_	no	- no covariates selected that represent potentially constraining environmental conditions (e.g. temperature)	W1_NET_	yes	- time allocation influenced by forage (landcover, ruggedness) and potentially constraining environmental conditions (snow, temperature, wind speed)
S2_NET_	no	- no covariates selected that represent potentially constraining environmental conditions (e.g. temperature)	W2_NET_	yes	- probability of resting increased with deep snow, low temperature, high wind speeds - long resting bout duration
S3_NET_	no	- time of day not selected as covariate	W3_NET_	no	- distinct daily scheduling of activities
S4_NET_	no	- year not selected as covariate	W4_NET_	partially	- year selected as covariate - no pronounced interannual variation in activity budgets

## Results

### State-allocation and goodness-of-model-fit

**Fig. 2:**
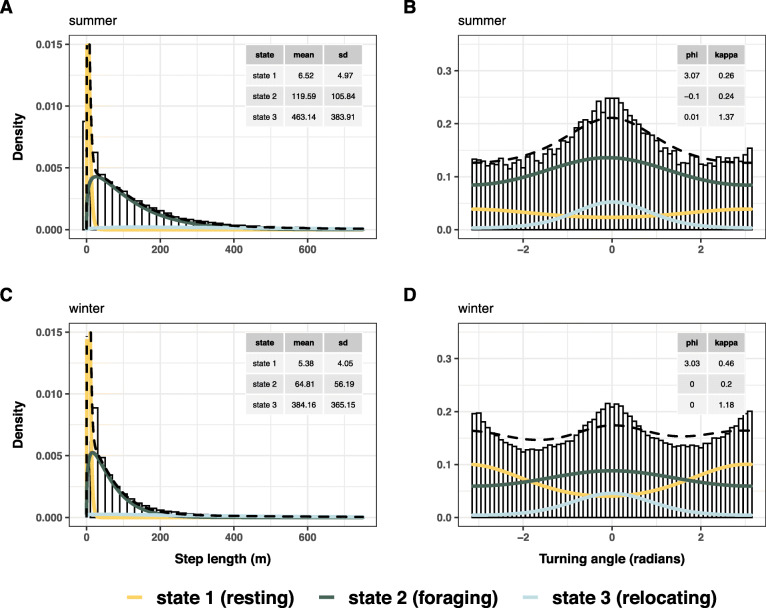
Histograms of step length and turning angle between hourly relocations, respectively, for the summer **a, b** and winter **c, d** season, overlaid with the state-dependent distributions as estimated by the HMMs selected by BIC. The state-dependent distributions were weighted according to the proportion of time spent in the different states, as inferred by the Viterbi sequence. Dashed black lines indicate the associated marginal observation distributions. Note that the x- and y-axes for step length were truncated at the upper range limit to facilitate visualisation (maximum observed step length was 3486 m for summer, and 3897 m for winter). Tables included in panels provide parameter estimates per state and model (mean step length with standard deviation; mean turning angle (phi) and angle concentration (kappa))

**Fig. 3:**
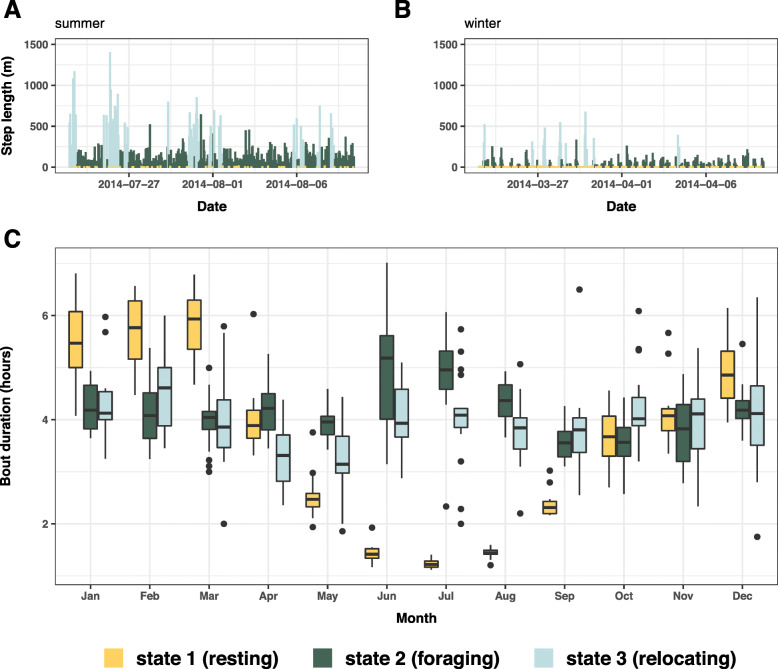
Example of state-decoded step lengths for the **a** summer and **b** winter season, showing a period of 18 days for one individual female, respectively. For all state-decoded muskox locations, see Additional file 1: Fig. S15. **c** Monthly boxplots for the individual-based mean duration of behavioural bouts

The estimated state-dependent distribution for step lengths and turning angles differed between summer and winter (Fig. 2), but states generally followed the same pattern: State 1 was characterised by short step lengths and high turning angles (i.e. undirected movements), presumed to represent resting behaviour. State 2 included medium step lengths and turning angles centred around zero but with low concentration (i.e. low kappa) around the mean (indicating tortuous movements with a slight overall tendency for forwards movements). State 3 was associated with larger step lengths and turning angles highly concentrated around zero, representative of very directed movements. We thus assumed states 2 and 3 to reflect foraging and relocating behaviour, respectively. This interpretation is corroborated by Fig. 3 a-b, displaying example time series of step lengths and associated decoded states for both seasons. Mean step lengths were generally smaller during winter than summer (Fig. 2), supporting the separate modelling of the two seasons. The model-induced marginal distributions of the two movement variables corresponded well with the underlying empirical distributions (Fig. 2). For more details on model evaluation, see Additional file 1: Figs. S8-S9. The forward covariate selection procedure produced different results for summer and winter (Additional file 1: Fig. S10).

### Behavioural time allocation during summer

**Fig. 4:**
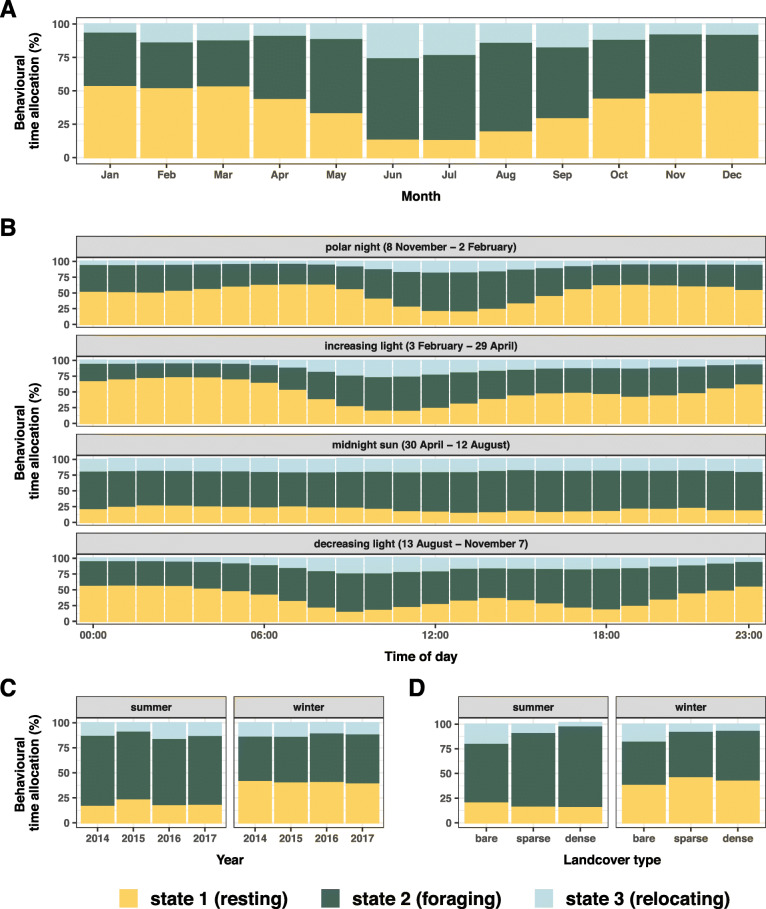
Behavioural time allocation in female muskoxen in northeast Greenland depending on **a** day of the year, aggregated by month, **b** time of day during different light seasons (polar night = period of 24 h of darkness, midnight sun = period of 24 h daylight), **c** year and **d** landcover type (bare ground, sparse or dense vegetation). Note that in **c** year t denotes the winter season t-1 to t, i.e. for instance 2014 is the winter 2013–2014. For behavioural time allocation by Julian day, i.e. not aggregated by month, see Additional file 1: Fig. S11 D

**Fig. 5:**
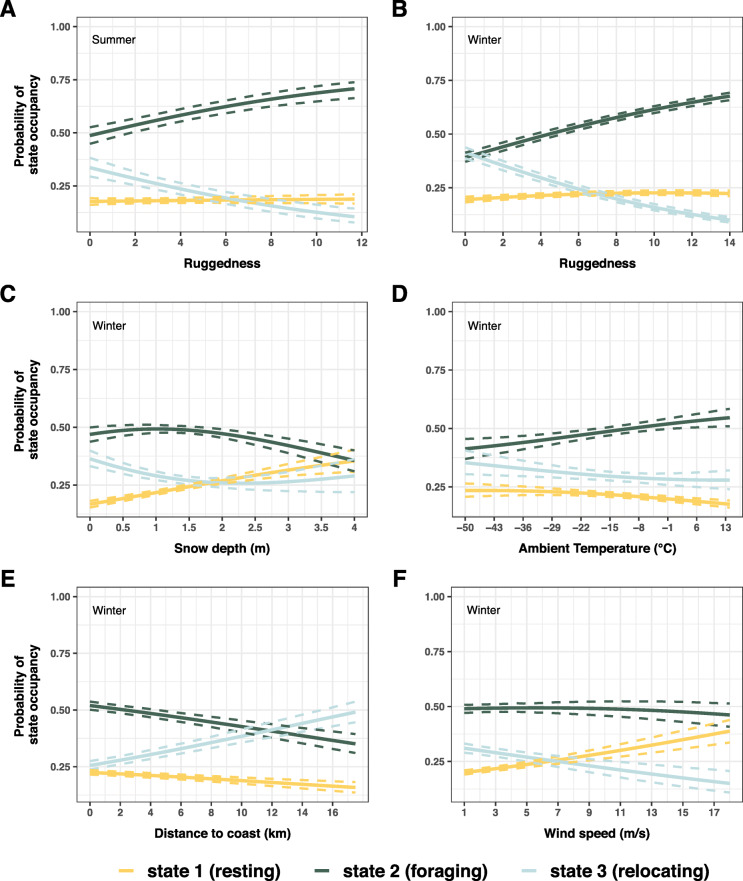
Stationary probabilities (mean and 95% CI) of behavioural state occupancy as a function of the environmental covariates included in the final HMMs for the **a** summer and **b-f** winter season. According to BIC model selection, the final summer model included light, landcover type, terrain ruggedness and Julian day as covariates; the final winter model included Julian day, time of day, landcover type, terrain ruggedness, snow depth, light, ambient temperature, year, distance to coast and wind speed. Probabilities were calculated for each covariate and state by fixing the values of the remaining continuous environmental covariates at their respective seasonal mean. Continuous temporal covariates were set to Julian Day 213 (i.e. August 1st) and 91 (i.e. April 1st) for summer and winter, respectively, and to12 o’clock for time of day. Categorical covariates were set to their corresponding reference categories, i.e. to bare ground (landcover type), daylight, and, for the winter model, winter 2013–2014 (year). Monte Carlo simulation from the estimator’s approximate multivariate normal distribution was used to obtain pointwise 95% CIs. Coefficients of the multinomial logistic regression underlying this figure, as well as figures for probabilities of behavioural state occupancy for different categories (e.g. sparse/dense vegetation, darkness), are provided in the supplementary materials (Additional file 1: Tables S2-S3, Figs. S12-S14)

For the summer season, the final model included light, landcover type, terrain ruggedness and Julian day as covariates (Additional file 1: Fig. S10), i.e. predominantly covariates reflecting variation in forage quality/quantity, supporting predictions S1_INTAKE_ and S2_INTAKE_ (Table 3). Muskox females spent an average of 69% [range: 63–74%] of time foraging, 19% resting [15–23%], and 12% relocating [7–19%] during summer (Additional file 1: Fig. S11 A), with the highest mean time spent foraging in August (66% [55–73%]), and only about 14% of time spent resting in June [10–21%] and July [10–17%] (Fig. 4 a). Time allocated to foraging was lowest on bare ground and highest in dense vegetation, and vice versa for relocating (Fig. 4 d). The probability of foraging increased with increasing terrain ruggedness (i.e. a proxy for vegetation heterogeneity, see Table 2), whereas probability of relocating decreased (Fig. 5 a). Neither landcover type nor terrain ruggedness strongly affected the probability of resting. Light seemed to have the strongest effect in summer, with muskoxen allocating more time to resting during darkness (32% [14–43%]) than daylight (18% [15–21%], Additional file 1: Fig. S11 B). Most observations included in the summer model were recorded during the midnight sun period, hence muskoxen were more likely to switch to the resting state during dark hours as soon as the sun began to set again (mid-August onwards). As evident from the covariate selection process and in line with predictions S3_INTAKE_ and S4_INTAKE_ (Table 3), neither time of day nor year explained much of the behavioural variation. Accordingly, no specific scheduling of daily activity could be detected during the midnight sun period (comprising most observations), and interannual differences in time allocation between summer seasons appeared minimal (Fig. 4 b-c).

### Behavioural time allocation during winter

The final winter model included Julian day, time of day, landcover type, terrain ruggedness, snow depth, light, ambient temperature, year, distance to coast and wind speed as covariates (Additional file 1: Fig. S10). In line with prediction W1_NET_, these covariates reflect variation in both forage as well as potentially constraining environmental conditions (Table 3). However, as time of day was selected as well, support for prediction W1_TIME_ was likewise indicated. The average proportion of time spent in the foraging state during winter was lower (45% [34–50%]), and time spent resting about twice as high as during summer (43% [37–3%]) (Additional file 1: Fig. S11 A). Time spent foraging dropped to lowest mean value of 34% in both February [20–46%] and March [19–45%], and time spent resting was highest in January (54% [46–62%]) (Fig. 4 a).

Similar to the summer model, covariates representing forage quality/quantity/accessibility (landcover, ruggedness, distance to coast, see Table 2) were positively related to the probability of foraging, and probability of relocating increased with decreasing probability of foraging. Muskoxen spent most time foraging in dense vegetation, and more time relocating on bare ground (Fig. 4 D). Increasing terrain ruggedness – in winter a proxy for heterogeneity in both snow accumulation and vegetation (Table 2) – increased the probability of foraging but decreased the probability of relocating (Fig. 5 b). Responses to environmental conditions constituting potential energetic constraints (snow depth, ambient temperatures, wind speed) generally supported prediction W2_NET_ (Table 3). When encountering deep snow, muskoxen were more likely to be resting, and less likely to be foraging or relocating (Fig. 5 c). Low ambient temperatures and high windspeeds likewise increased the probability of resting (Fig. 5 d, f). As opposed to the midnight sun period, distinct daily activity patterns were observed for the rest of the year (Fig. 4 b). During the polar night and the period of increasing light, pronounced unimodal activity peaks were apparent, whereas a bimodal pattern was exhibited during the period of decreasing light. These results are in accordance with prediction W3_TIME_ (Table 3). Although year was selected as covariate, activity budgets did not show pronounced interannual variation between winter seasons (Fig. 4 c), lending some support for both W4_INTAKE_ and W4_NET_ (Table 3).

### Duration of behavioural bouts

Throughout the year, duration of foraging and relocating bouts remained relatively constant (foraging (mean [range]) = 4.2 h [3.5–5 h]; relocating = 4 h [3.2–4.6 h]) (Fig. 3 c). Resting bout duration, however, varied markedly over the course of the year (mean = 3.6 h), with longest bouts in March (5.8 h [4.7–6.8 h]) and shortest in July (1.2 h [1.1–1.4 h]) (Fig. 3 c). This result is indicative of S1_INTAKE_ and either W1_INTAKE_ or W2_NET_ (Table 3).

## Discussion

Understanding how environmental conditions shape the foraging behaviour of free-ranging animals is a fundamental aspect of ecology, as foraging tactics influence individuals’ fitness and thus ultimately population dynamics [42, 43]. However, assessing animals’ foraging strategies and testing their optimality over time and space is challenging, especially for species where detailed year-round observations and direct assessments of foraging efforts and gains are not feasible. Muskoxen inhabit one of the most remote and seasonally extreme environments of this planet, and our study provides the first detailed account of behavioural strategies covering the full seasonal cycle over multiple years with pronounced variation in environmental conditions.

### Optimising energy intake? Foraging strategies during summer

As capital breeders [44], muskoxen rely heavily on body reserves gained during the short forage-abundant summer to secure winter survival and calf production [45, 46] – even more so than reindeer/caribou (*Rangifer tarandus*), the only other arctic ungulate species [44]. In agreement, we found muskox time allocation during summers to be influenced primarily by forage and light conditions. Activity budgets indicated almost continuous foraging (Fig. 4 a), interrupted only by short resting and relocating periods (Fig. 3 c), with no specific daily scheduling of activities (Fig. 4 b) and little interannual variation (Fig. 4 c). These results clearly indicate that muskoxen in northeast Greenland follow an energy intake maximisation strategy during summer, as previously suggested by studies based on direct behavioural observations for other muskox populations [47–49].

Muskoxen allocated substantially more time to foraging during summer than winter (Fig. 4 a, Additional file 1: Fig. S11 A), mainly as a result of drastically shorter resting bouts (Fig. 3 c). This seems to be a widespread behavioural pattern observed in muskox populations elsewhere [3, 49], but also other ungulate species [2, 4, 50]. As high digestibility of plant material decreases time required for rumination [50], the short resting bouts likely reflect abundant high-quality forage. Indeed, digestibility and quality of summer forage is generally high in the Arctic [49, 51], and terrain heterogeneity and associated differences in plant phenology allow for selective foraging of vegetation in early growth stages (i.e. with highest digestibility) until late in the growing season [49]. The positive effect of terrain ruggedness on foraging activity (Fig. 5 a) was equally evident from our results.

Heat stress is a well-known constraint to animals’ activity [8, 32], especially in cold-adapted species [4]. Although muskoxen may be susceptible to high temperatures, physiological and behavioural limits to heat tolerance are unclear [52]. According to our results, muskox foraging behaviour appears unconstrained by heat stress given the experienced summer temperatures (up to 16.4 °C).

### Digesting or conserving energy? Foraging strategies during winter

While arctic ungulates may heavily rely on body reserves gained during summer, foraging strategies during the long winters have to supplement summer reserves and may consequently be likewise important for survival [53], foetus development [54] and maintenance of the rumen microbiome [55]. We found partial support for all three tested OFT strategies (Table 3), hence our results are not easily reconcilable with just one dominant foraging strategy.

Evidently, muskoxen were less active (i.e. foraging or relocating) and rested more during winter (Fig. 4 a, Additional file 1: Fig. S11 A), with considerably longer resting bouts (Fig. 3 c). This increase in resting time is likely a functional response to the reduction in forage quality, requiring more time for digestion [50], providing some support for an energy intake maximisation strategy. As in summer, forage conditions (i.e. landcover type, ruggedness) strongly influenced behavioural time allocation in winter, with suboptimal foraging conditions apparently motivating relocation to different areas (Fig. 5 b).

However, behavioural time allocation and state-switching were also, albeit to a lesser degree, influenced by environmental conditions reflecting potential energetic constraints: Muskoxen reduced foraging activity and were more likely to rest when encountering conditions leading to heat loss (low ambient temperatures, high wind speeds, Fig. 5 d, f) or increased costs of foraging and movement (deep snow, Fig. 5 c). This finding, suggesting attempts to conserve energy, is indicative of a net energy maximisation strategy and rather contradicts a strategy of energy intake maximisation_._ Similar responses to adverse weather conditions have been found for muskoxen in West Greenland [47] as well as other ungulate species [2, 4], and inactivity and resting while lying down have been shown to reduce the metabolic costs of thermoregulation in cold weather [56]. However, given our movement data at an hourly resolution, we are currently not able to distinguish ‘true’ resting (i.e. time that would otherwise be available for foraging) from ruminating (dictated by digestive constraints). To determine to what degree ruminants living in highly seasonal environments allocate time to digestion versus energy conservation, future studies should attempt to more finely distinguish these two behaviours.

### Diel activity patterns

Throughout the year, muskoxen were active during all hours of the day, but more likely to rest during darkness (Fig. 4 b, Additional file 1: Fig. S11 B). At the latitude of our study area (74 ° N), distinct cycles of daylight and darkness only occur for 12.5 weeks in spring and autumn, respectively. Nonetheless, we found distinct scheduling of daily activity for all seasons except for the period of continuous daylight (Fig. 4 b). However, predation risk and human disturbance are extremely low in our study area [26], and we therefore consider these factors unlikely determinants of activity scheduling. Hence, we argue against time minimisation as dominant foraging strategy in this population. Instead, we interpret these results as a clear expression of basic ultradian ruminant activity patterns, as also observed in predator-free Svalbard reindeer at similar latitudes [2, 57]. Moreover, time spent resting increased steadily from the end of summer towards highest levels in mid-winter (January to March), with a concomitant decrease in foraging time (Fig. 4 a). As the quality of muskox diets in the study area decreases significantly over winter [30], this result provides additional evidence against a time minimising strategy.

### Limitations and future prospects

Previous studies assessing muskox activity patterns based on direct behavioural observations [3, 36, 47–49, 58] were of small geographic coverage and biased towards summers and daylight hours. Coupling telemetry data with hidden Markov modelling, we overcome these observational challenges, allowing us to gain detailed year-round insights into this key arctic herbivore’s behavioural variation. Our quantification of season-specific time allocation and identification of foraging constraints constitutes a critical step towards direct tests of optimal foraging strategies [32]. Clearly, combining location data with more precise estimates of availability, quality and type of consumed forage allows for a more direct assessment of obtained energetic benefits and thus foraging strategies [12], and hence constitutes a logical next step to complement our approach.

As we used an unsupervised HMM approach based on the distribution of step lengths and turning angles and not validated by direct observations of the animals, state classification should be interpreted with caution, i.e. only be considered as proxy for the underlying ‘true’ animal behaviour [59]. We limited our classification to the three most common, straightforward to interpret behavioural states, as recommended in an unsupervised HMM framework [37]. The state characterisations corresponded well to those described for other large herbivores [60, 61]. The resting state reflected minimal movements and uniformly distributed turning angles (with a somewhat higher probability of being directed towards 180° due to an artefact of GPS error). The foraging state was associated with slow, often tortuous movements resulting from alternate stationary foraging and intermittent small relocations when searching for suitable foraging spots. The relocation state was characterised by the longest steps and highest directional persistence. Muskoxen specifically have been found to move between 1.6 and 5.8 m/min while foraging, depending on season and vegetation type encountered [47]. Aggregated to an hourly scale, these values correspond well to the HMM-derived distribution of step lengths in state 2 (i.e. foraging) for summer and winter (Fig. 2). Obviously, muskoxen express a more nuanced range of behaviours than the three behavioural states considered here. However, due to the hourly resolution of our data, many behaviours can fall ‘under the radar’, i.e. the model-derived behavioural state classification can only provide a rough estimate of hourly behaviour and can generally not account for short-term or non-exclusive behaviours, such as social interactions or short foraging ‘pit-stops’ during larger-scale relocation bouts.

Nonetheless, even the interpretation of a 3-state model provides challenges. For example, muskoxen typically did not move extensively within an hour (see Fig. 2), making the distinction between resting and foraging behaviour difficult at times. Misclassifications may for instance happen during winter when the necessity to crater through snow to reach the underlying vegetation limits displacement during foraging, leading to an overestimation of resting time. This may have contributed to the fact that we were unable to determine a dominant fitting OFT strategy for winter. However, cratering behaviour appears confined to rather shallow snow depths [62], and muskoxen were found to rest longer as snow thickness at feeding craters increased [58]. These observations support the overall trend of decreasing foraging probability with increasing snow depth in our study. In summer, when switching between resting and foraging is highly volatile, short resting bouts (i.e. below 1 h) in particular may be masked, potentially leading to an overestimation of foraging time. Overall, however, we are confident that state classification in our HMMs is robust and captures most of the variation in muskox movement behaviour. A validated supervised classification approach would obviously help to quantify and/or rule out uncertainties related to misclassification, but was not feasible in the context of our study as direct observations were not possible in winter, i.e. for most of the year. Future studies could further refine state estimation by coupling GPS location data with more fine-scaled, continuous acceleration or other biologging data using hierarchical HMMs [63].

While our ability to collect fine-scale movement data is rapidly increasing, such data needs to be matched with environmental data at relevant temporal and spatial scales. Here, we tested a wide range of covariates, including rarely available temporally dynamic and spatially explicit covariates at the highest spatial resolution (300 m) and temporal frequency (3 h and daily) possible for this region. Although these covariates should in theory most directly represent the conditions influencing individuals and consequently explain much of the behavioural variation, this was only partially reflected in the results of the covariate selection process. This could signal a real biological effect, suggesting that muskoxen, well-adapted to extreme weather variability, are not particularly sensitive to dynamically changing environmental conditions. It may, however, also indicate that in highly heterogeneous environments, temporally static proxies for resource and climate gradients at high spatial resolution (e.g. landcover, ruggedness) may outperform temporally dynamic direct variables at coarser scales (e.g. NDVI). Given the importance of vegetation and snow conditions for alpine and arctic ungulates, further improvements in vegetation mapping as well as observations and modelling of wildlife-relevant snow variables [20, 35] are critical for detailed assessments of how animals respond to environmental variability.

## Conclusions

By combining unique data sets of GPS-based movements and spatiotemporal environmental covariates within HMMs, we tested a series of qualitative predictions derived from upscaled OFT. We conclude that during the brief high-arctic summers, muskox females adopt an energy intake maximisation strategy, largely unconstrained by environmental conditions. For the long winter season, our results indicate partial support for all three tested foraging strategies. However, deep snow, low ambient temperatures and strong winds were clearly constraining foraging behaviour in winter (Fig. 5), with muskoxen instead allocating more time to resting, likely to conserve energy.

With climate change altering seasonal patterns and thus challenging species’ behavioural rhythms and adaptations [64, 65], assessing how environmental conditions shape the foraging behaviour of free-ranging animals is an increasingly urgent task. In environments as marginal as the high Arctic, where rapid warming is already impacting the entire biophysical system [66], even small deviations from optimal foraging behaviour may potentially have large consequences for reproductive success and survival, and thus ultimately population dynamics. Providing novel insights into the current relationship between environmental conditions and muskox behaviour, this study improves our understanding of how ungulate species are able to survive under highly variable conditions via adaptive fine-scale behaviour and lays the foundation for assessing behavioural plasticity for future adaptation.

## Supplementary information

**Additional file 1.** An application of upscaled optimal foraging theory using hidden Markov modelling: year-round behavioural variation in a large arctic herbivore.

## Data Availability

The datasets supporting the conclusions of this article are available in the Zenodo repository, 10.5281/zenodo.3768080.

## Abbreviations

BIC: Bayesian Information Criterion
GPS: Global Positioning System
HMM: hidden Markov model
NDVI: Normalized Difference Vegetation Index
OFT: Optimal foraging theory
