# Supplementary material for: An application of upscaled optimal foraging theory using hidden Markov modelling: year-round behavioural variation in a large arctic herbivore
Source: Mov Ecol. 2020 Jun 5;8:25. doi: 10.1186/s40462-020-00213-x (PMC7275509; doi:10.1186/s40462-020-00213-x)
Supplement: Supplementary file 1 — Additional file 1. An application of upscaled optimal foraging theory using hidden Markov modelling: year-round behavioural variation in a large arctic herbivore. [file 40462_2020_213_MOESM1_ESM.pdf]

Supplementary materials

An application of upscaled optimal foraging theory using hidden Markov modelling: year-round behavioural variation in a large arctic herbivore

Larissa T. Beumer[1,2,*], Jennifer Pohle[3], Niels M. Schmidt[1,2], Marianna Chimienti[1,2], Jean-Pierre Desforges[1,2,4], Lars H. Hansen[1,2], Roland Langrock[3], Stine Højlund Pedersen[5,6], Mikkel Stelvig[7], Floris M. van Beest[1,2]

[1] Department of Bioscience, Aarhus University, 4000 Roskilde, Denmark

[2] Arctic Research Centre, Aarhus University, 8000 Aarhus C, Denmark

[3] Department of Business Administration and Economics, Bielefeld University, 33615 Bielefeld, Germany

[4] Natural Resource Sciences, McGill University, Ste Anne de Bellevue, Quebec H9X 3V9, Canada

[5] Cooperative Institute for Research in the Atmosphere, Colorado State University, Fort Collins, CO 80523, USA

[6] Department of Biological Sciences, University of Alaska Anchorage, Anchorage, AK 99508, USA

[7] Copenhagen Zoo, 2000 Frederiksberg, Denmark

* Corresponding author: larissa.beumer@bios.au.dk

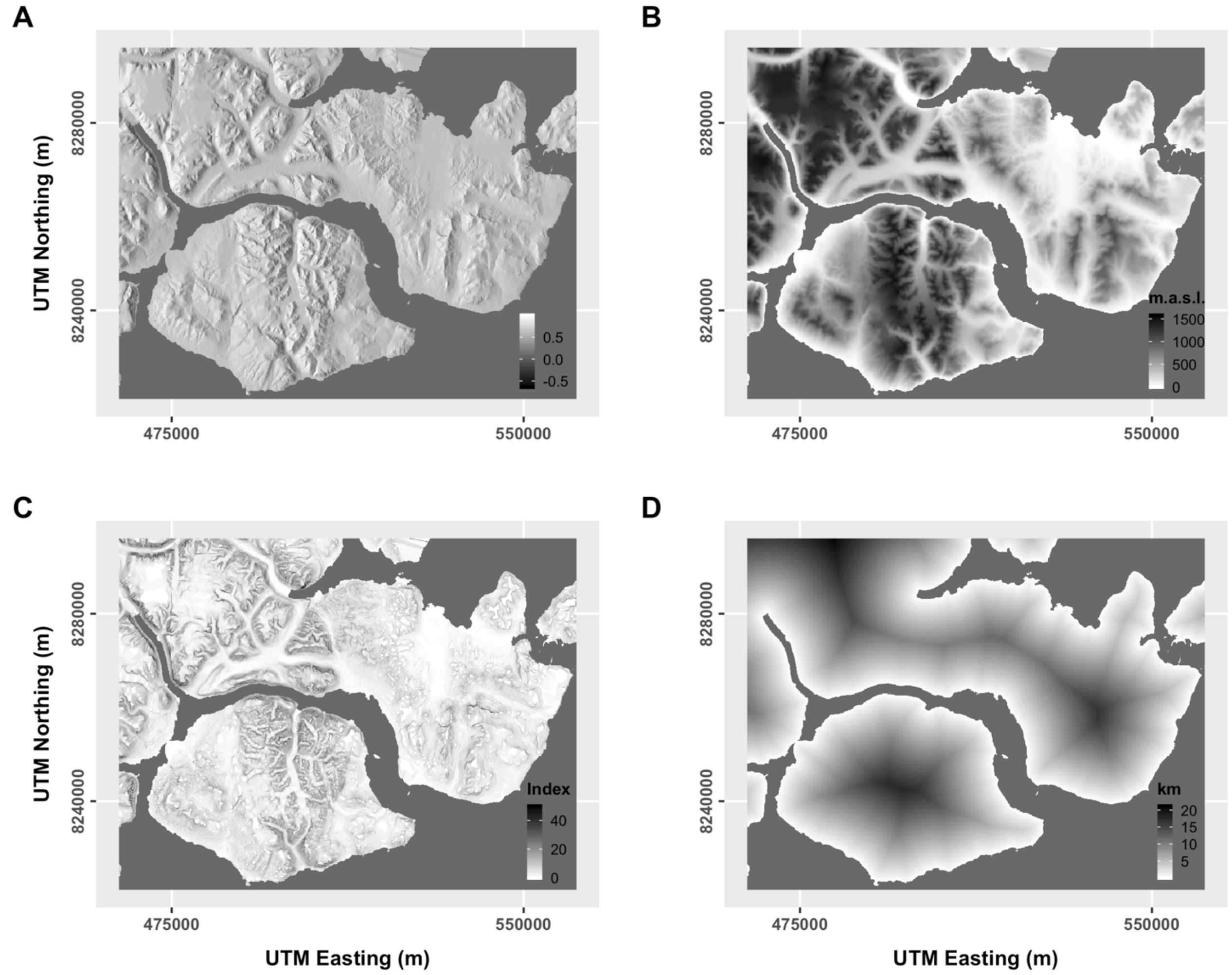

Figure S1: Distribution of static covariates across the study area (WGS 84, UTM zone 27): (A) ‘hillshade’ (unitless), (B) elevation (meters above sea level), (C) terrain ruggedness (index) and (D) distance to coast (km). Dark grey background colour indicates ocean areas.

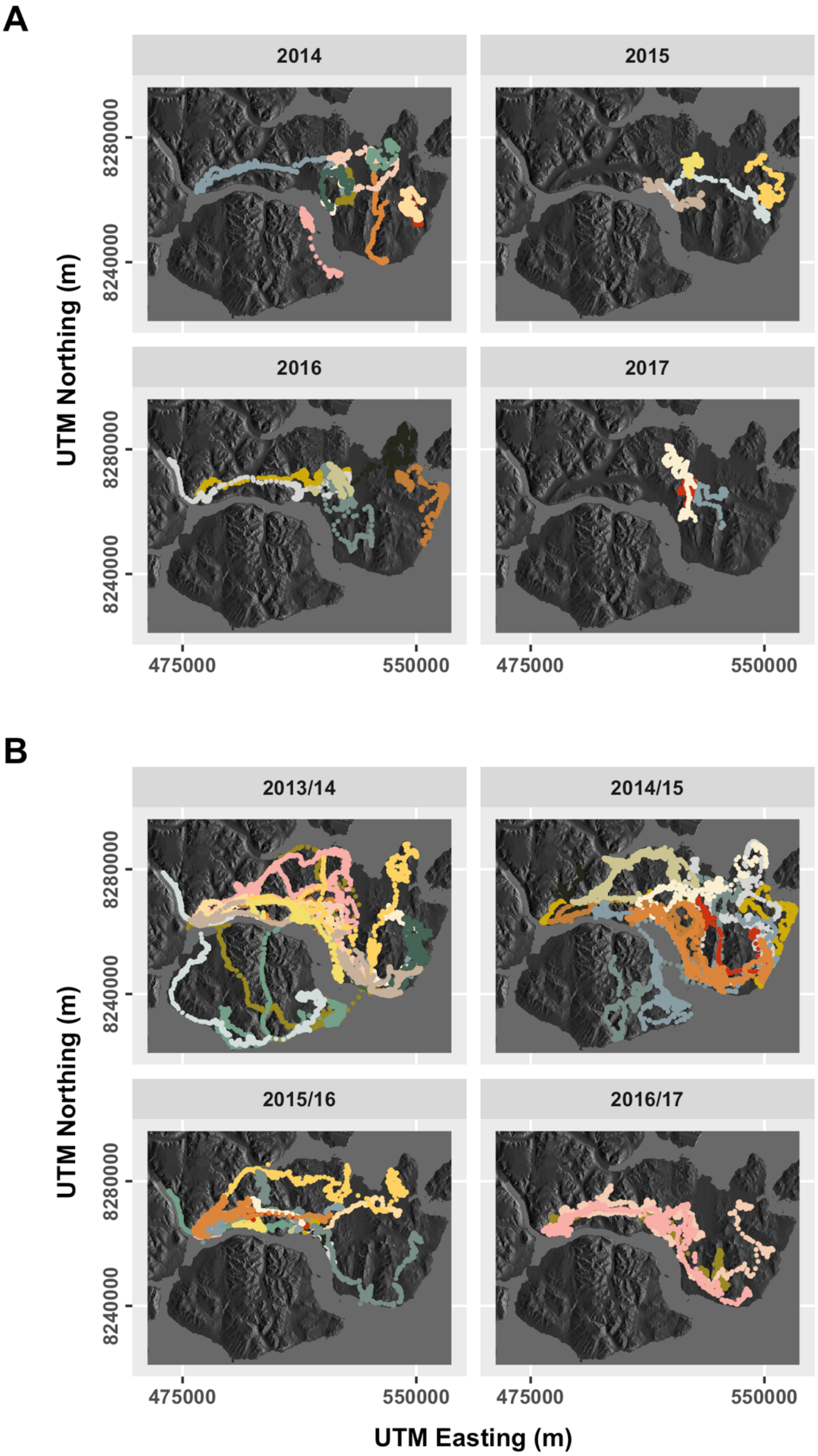

Figure S2: Muskox observations classified as either (A) snow-free summer or (B) snow-covered winter bursts, colour-coded by burst ID per season.

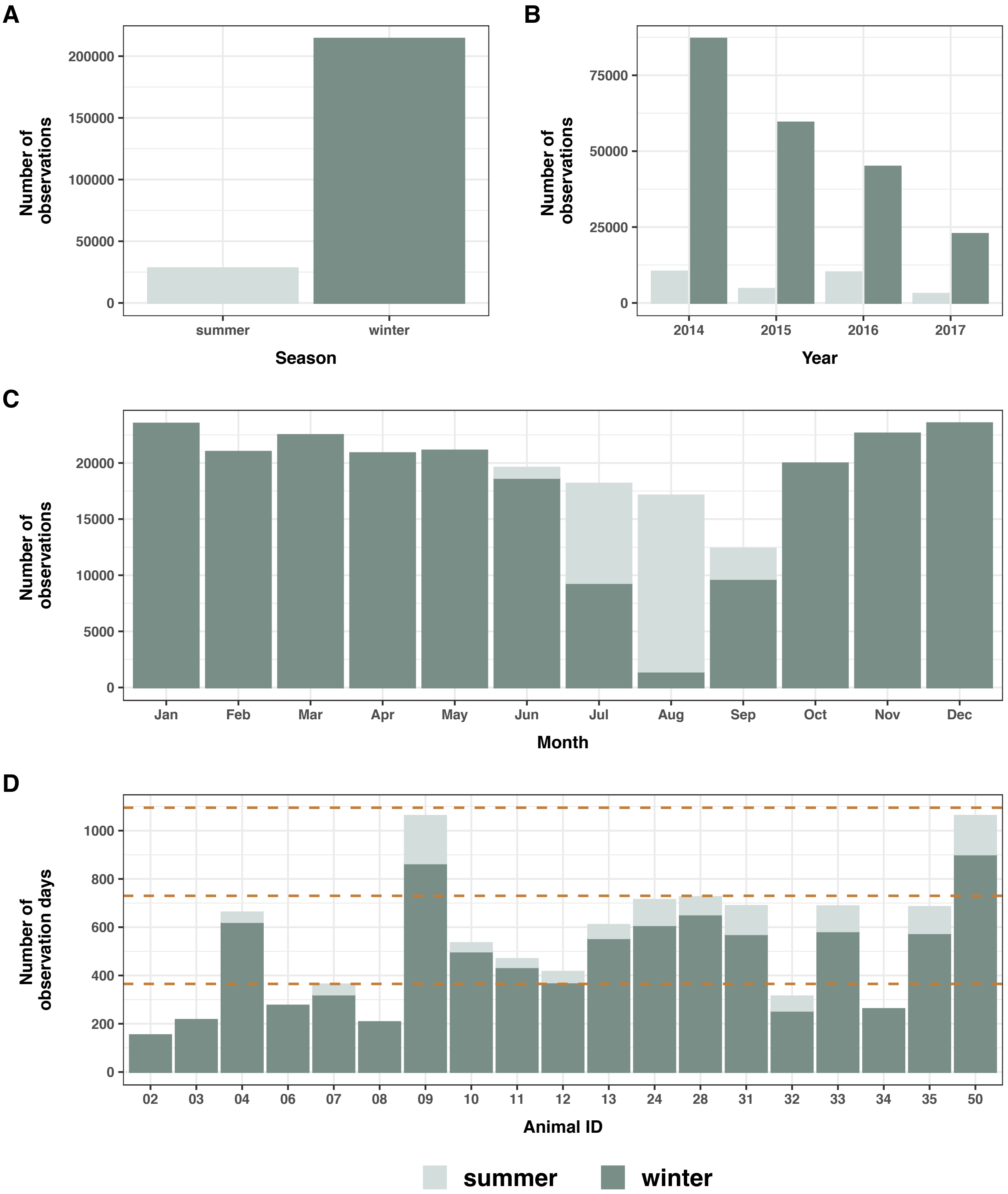

Figure S3: Overview of muskox observations (n = 242378) included in this study. Number of observations (A) included in the Hidden Markov model for the snow-free summer and the snow-covered winter season, respectively, (B) detailed per season and year and (C) per month. (D) Number of observation days per animal, i.e. female adult muskox included in the study (n = 19). Brown dashed lines indicate 365, 730 and 1095 days (i.e. 1, 2 and 3 years) of tracking data, respectively. Note that in (B) year t denotes the winter season t-1 to t, i.e. for instance 2014 is the winter 2013-2014.

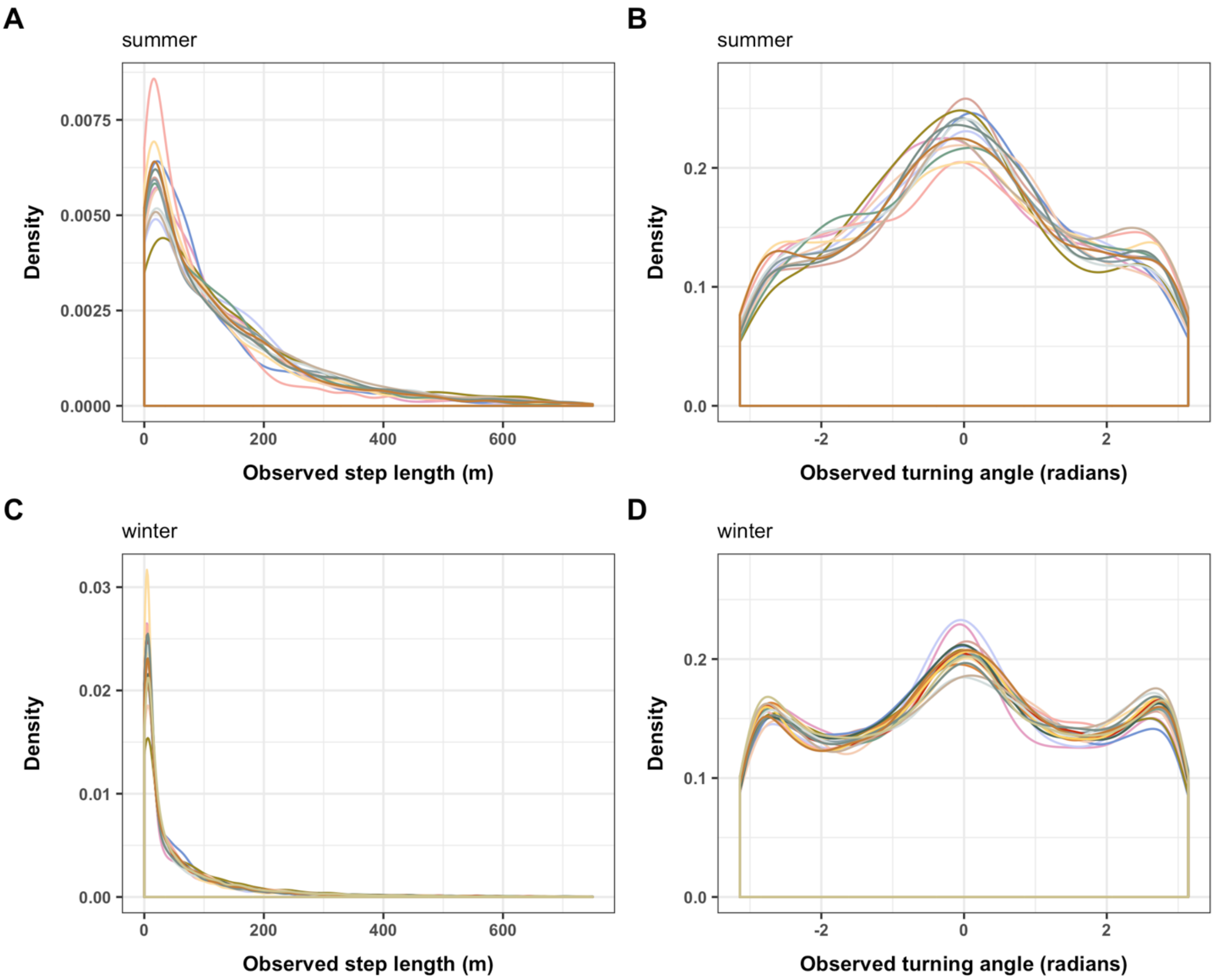

Figure S4: Individual variation in movement variables (kernel density estimates of observed step lengths and turning angles) during the snow-free summer (A-B) and the snow-covered winter season (C-D).

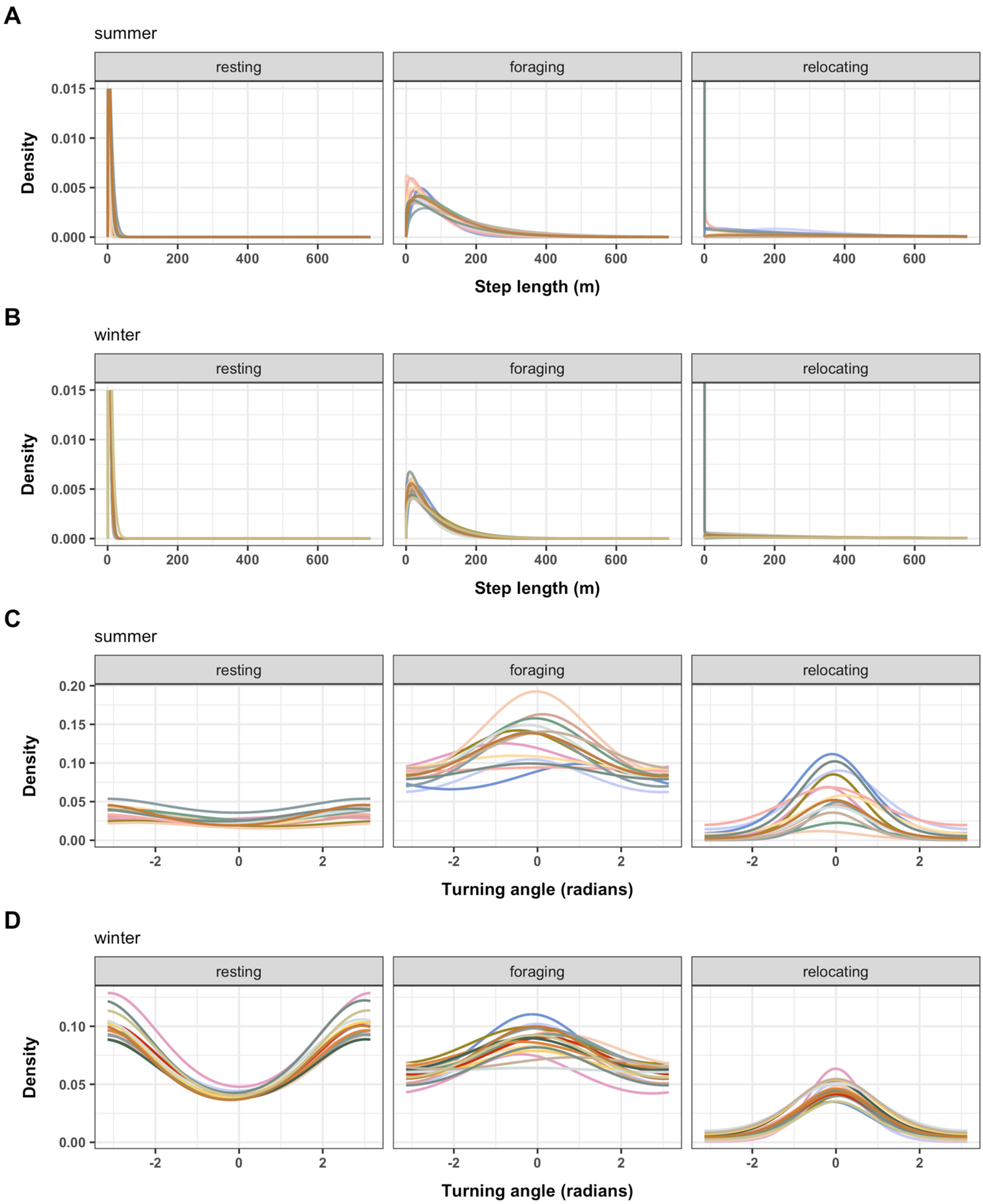

Figure S5: Individual variation in state-dependent distributions for step length and turning angles, derived from 3-state HMMs, fitted separately for each animal/season. For each model, 10 sets of random starting values were tested; results are shown for the model with highest log-likelihood values. Models did not include covariates.

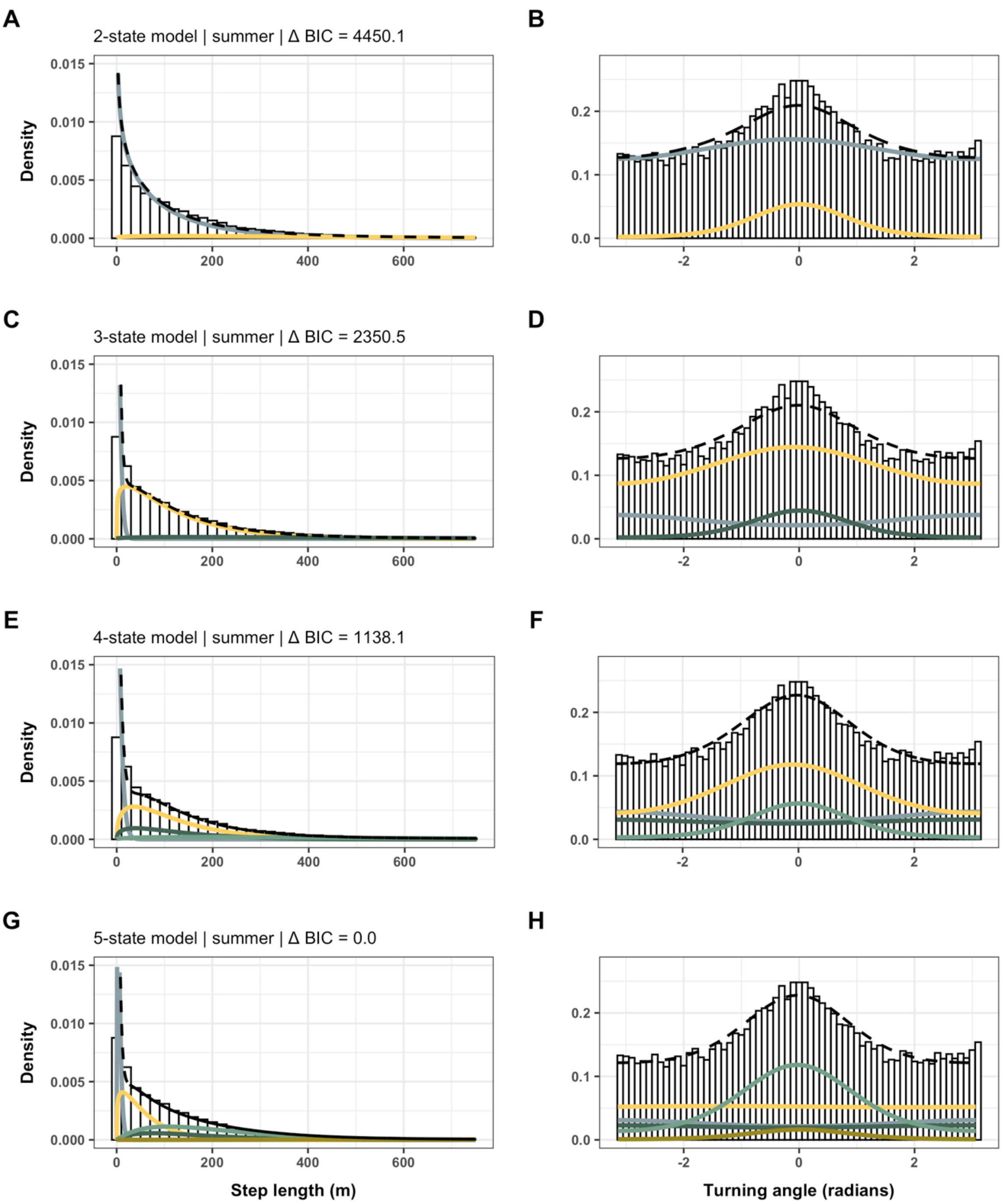

Figure S6: Histograms of step length and turning angle between hourly relocations, respectively, for the summer season, overlaid with the state-dependent distributions as estimated by initially explored HMMs including 2 (A-B), 3 (C-D), 4 (E-F) and 5 (G-H) states without covariates. We tested 10 sets of starting values for each model, respectively. The state-dependent distributions were weighted according to the proportion of time spent in the different states, as inferred by the Viterbi sequence. Dashed black lines indicate the associated marginal observation distributions. Note that the x- and y-axes for step length were truncated at the upper range limit to facilitate visualisation (maximum observed step length was 3486 m). Delta BIC values (differences between the respective model and the best-ranked model) are provided in panel titles for model comparison.

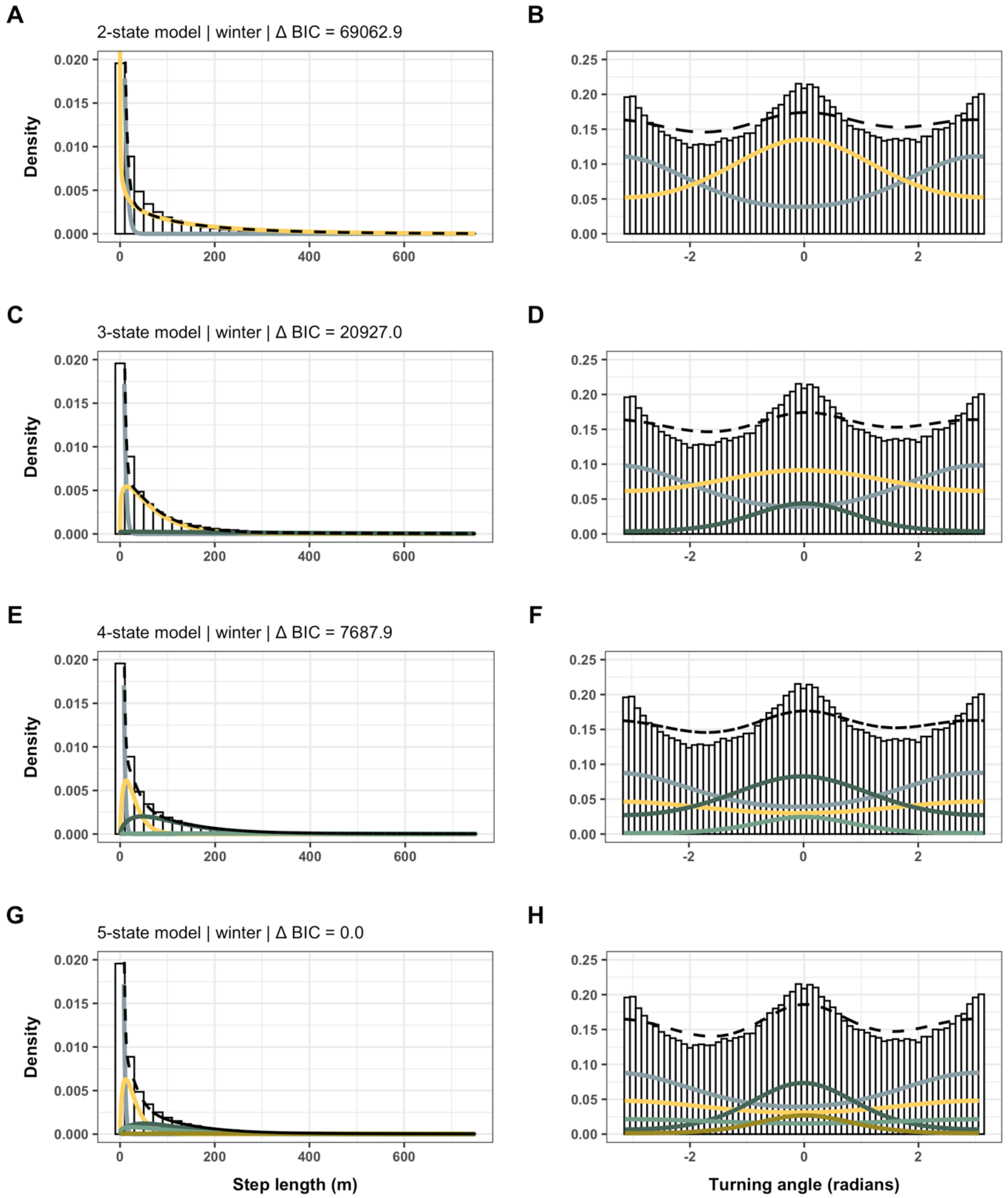

Figure S7: Histograms of step length and turning angle between hourly relocations, respectively, for the winter season, overlaid with the state-dependent distributions as estimated by initially explored HMMs including 2 (A-B), 3 (C-D), 4 (E-F) and 5 (G-H) states without covariates. We tested 10 sets of starting values for each model, respectively. The state-dependent distributions were weighted according to the proportion of time spent in the different states, as inferred by the Viterbi sequence. Dashed black lines indicate the associated marginal observation distributions. Note that the x- and y-axes for step length were truncated at the upper range limit to facilitate visualisation (maximum observed step length was 3897 m). Delta BIC values are provided in panel titles.

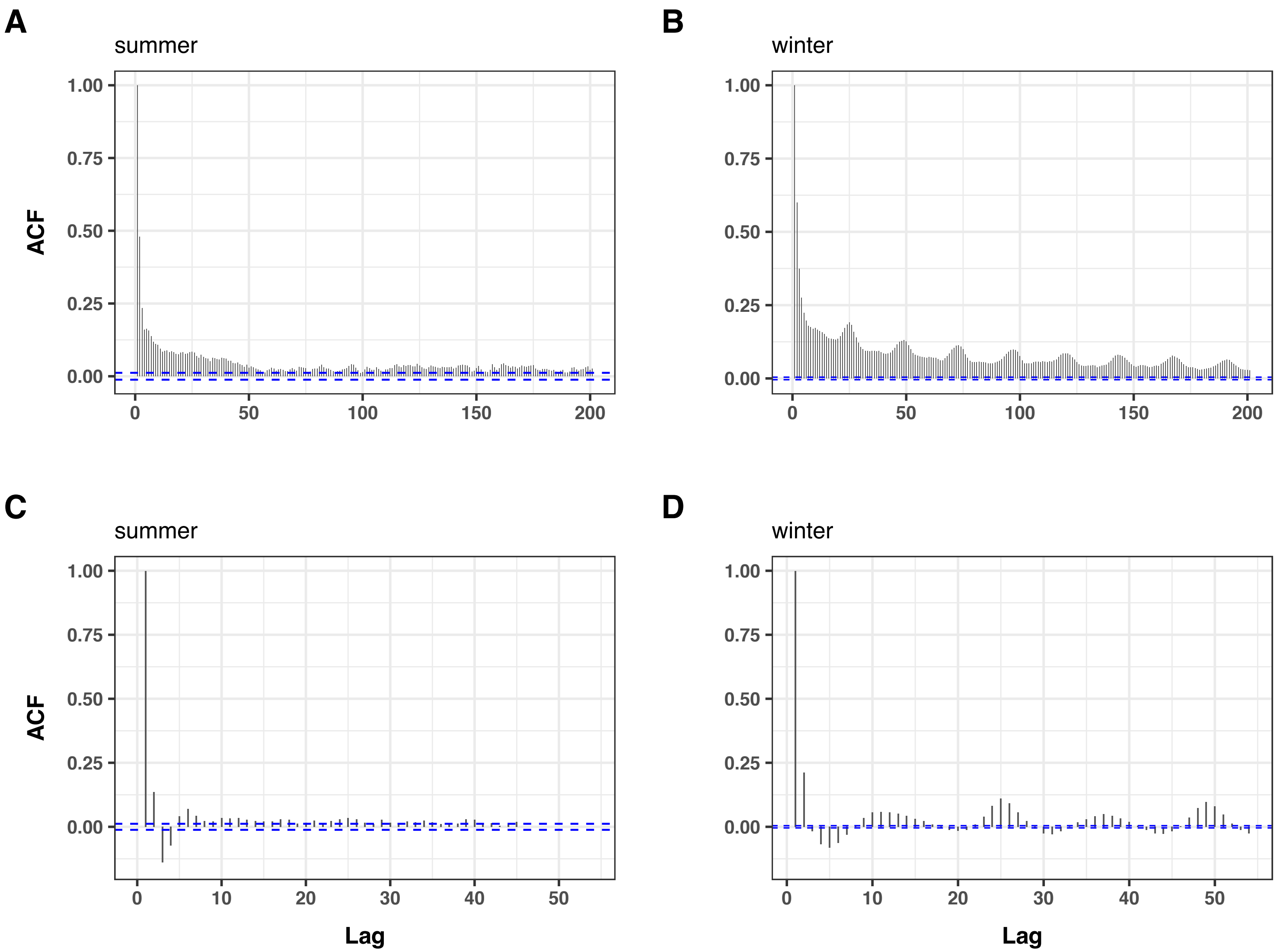

Figure S8: Autocorrelation structure in step length for track bursts classified as (A) summer (snow-free) and (B) winter (snow-covered), as well as in pseudo-residuals for the movement variable step length included in the three-state bivariate HMMs fitted to the muskox movement data for the (C) summer and (D) winter season (for the best model according to BIC, respectively).

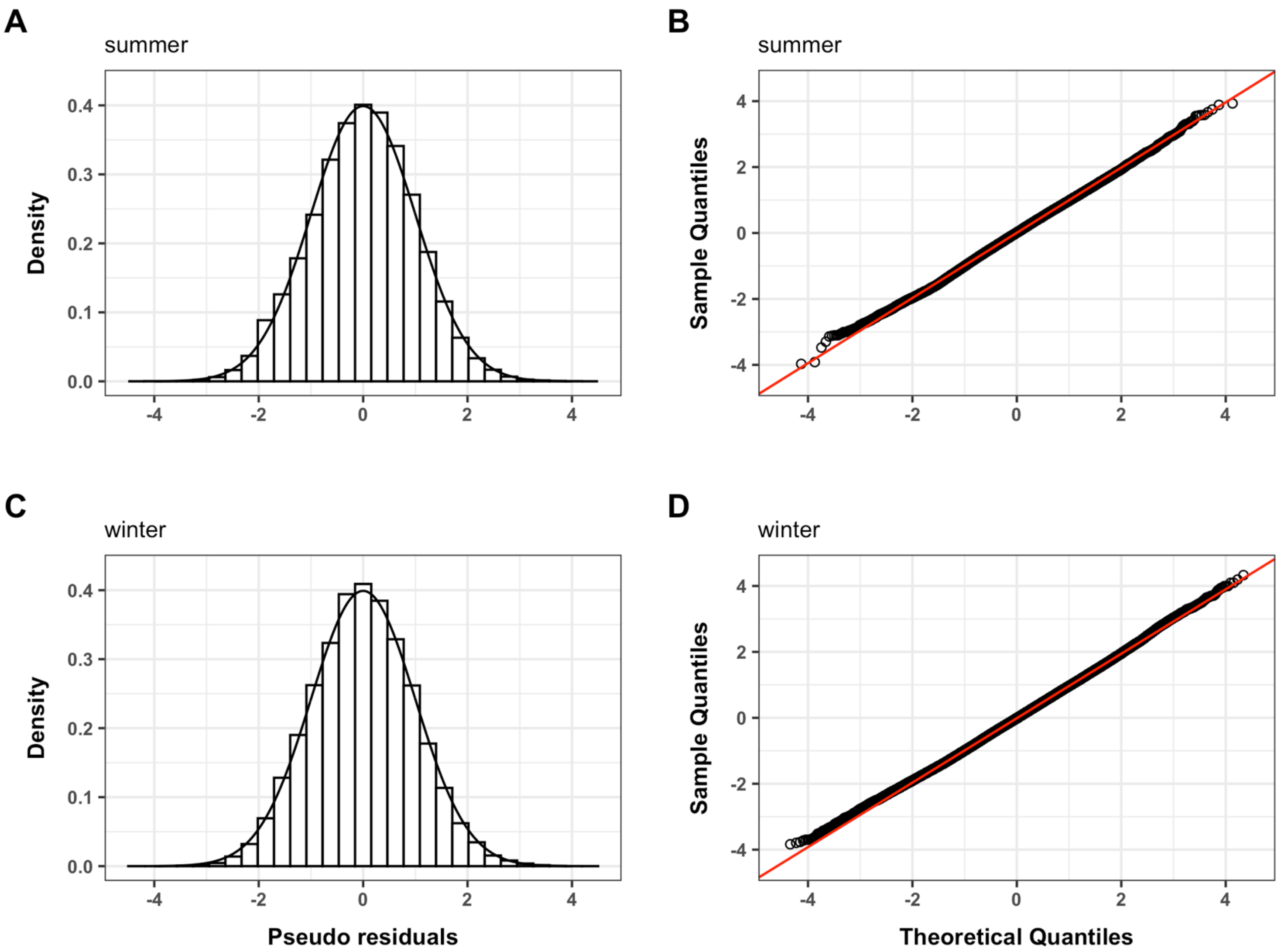

Figure S9: Distribution (A, C) and quantile-quantile (B, D) plots of the pseudo-residuals for the movement variable step length included in the three-state bivariate HMMs fitted to the muskox movement data for the summer (snow-free) (A, B) and winter (snow-covered) (C, D) season, for the best models according to BIC.

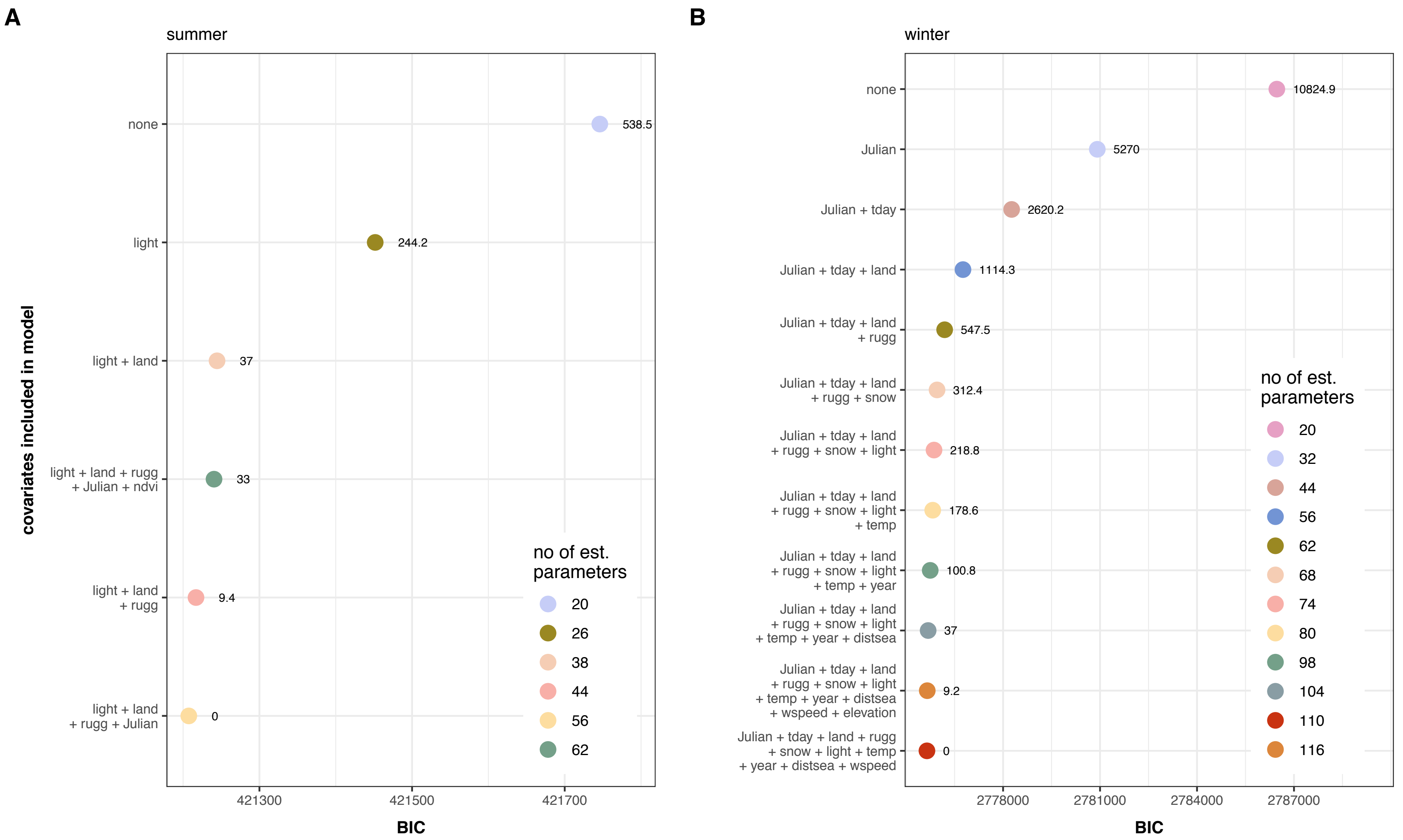

Figure S10: Results of the forward model selection process to determine the influence of a total of 14 covariates considered in the HMMs for the (A) summer and (B) winter season, respectively. Covariate selection was based on the Bayesian Information Criterion (BIC). Point labels correspond to differences in BIC values between the respective model and the best-ranked model (i.e. deltaBIC).

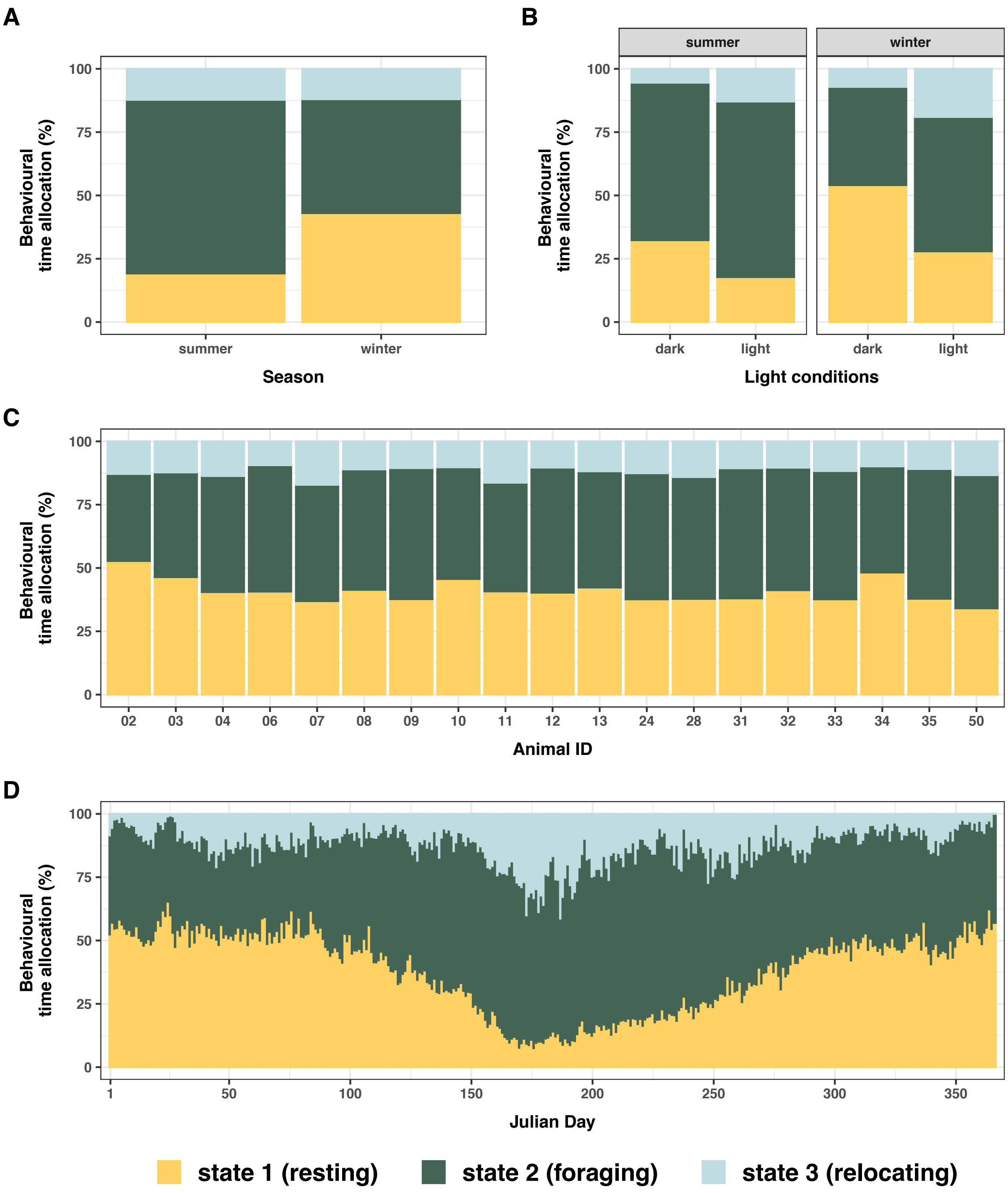

Figure S11: Behavioural time allocation in female muskoxen in northeast Greenland depending on (A) season, (B) light conditions (i.e. daylight or darkness at time of observation) for the summer and winter season, respectively, (C) animal ID and (D) Julian Day (i.e. day of the year).

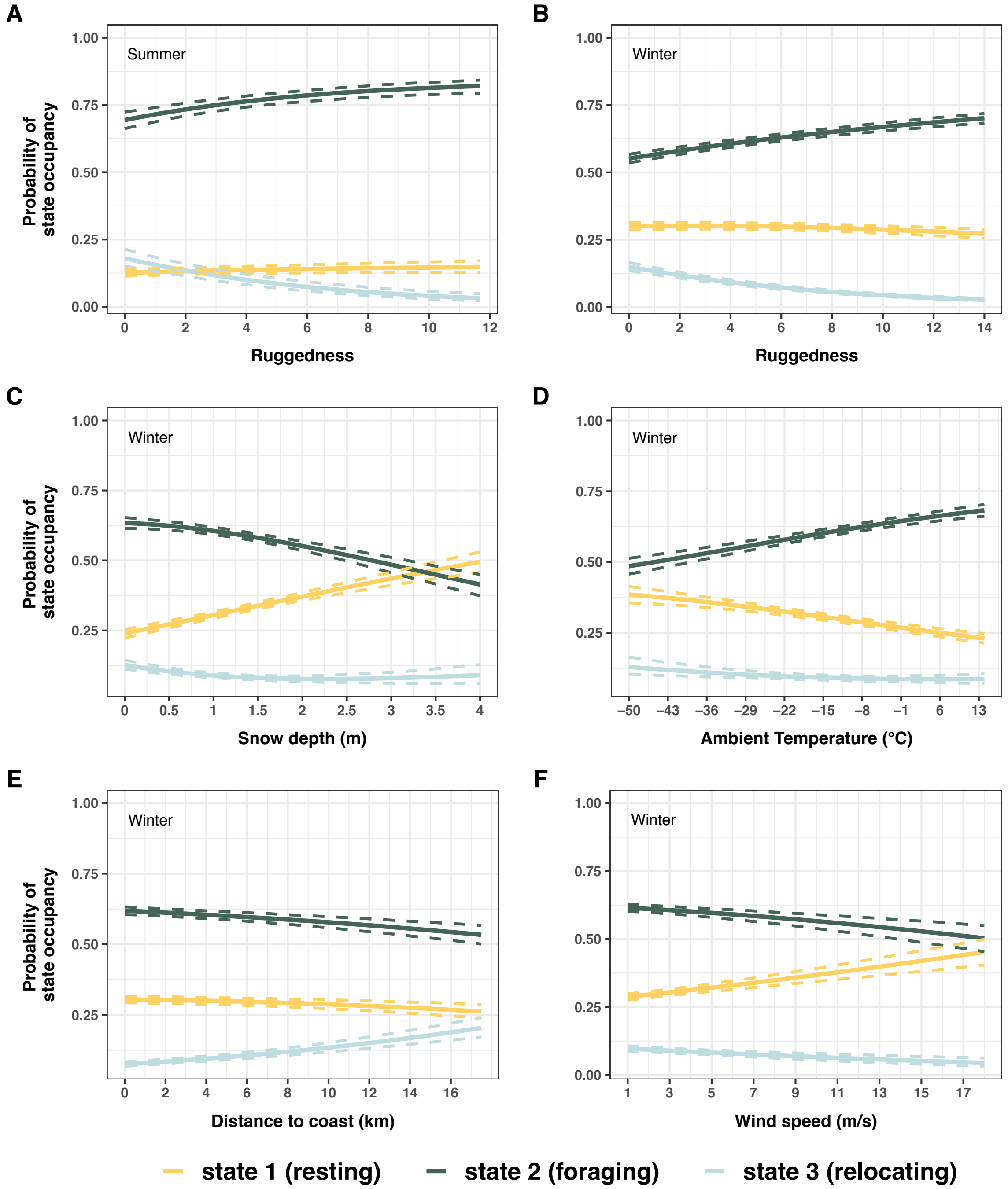

Figure S12: Probabilities (mean and 95% CI) of behavioural state occupancy as a function of the environmental covariates included in the BIC-selected bivariate HMMs for the (A) summer and (B-F) winter season. Probabilities were calculated for each covariate and state by fixing the values of the remaining continuous environmental covariates at their respective seasonal mean. Continuous temporal covariates were set to Julian Day 213 (i.e. August 1st) and 91 (i.e. April 1st) for summer and winter, respectively, and to12 o'clock for time of day. Landcover type was set to **sparse vegetation**, and the remaining categorical covariates to their corresponding reference categories (i.e. daylight, and winter 2013-2014 for winter model). Monte Carlo simulation from the estimator's approximate multivariate normal distribution was used to obtain pointwise 95% CIs.

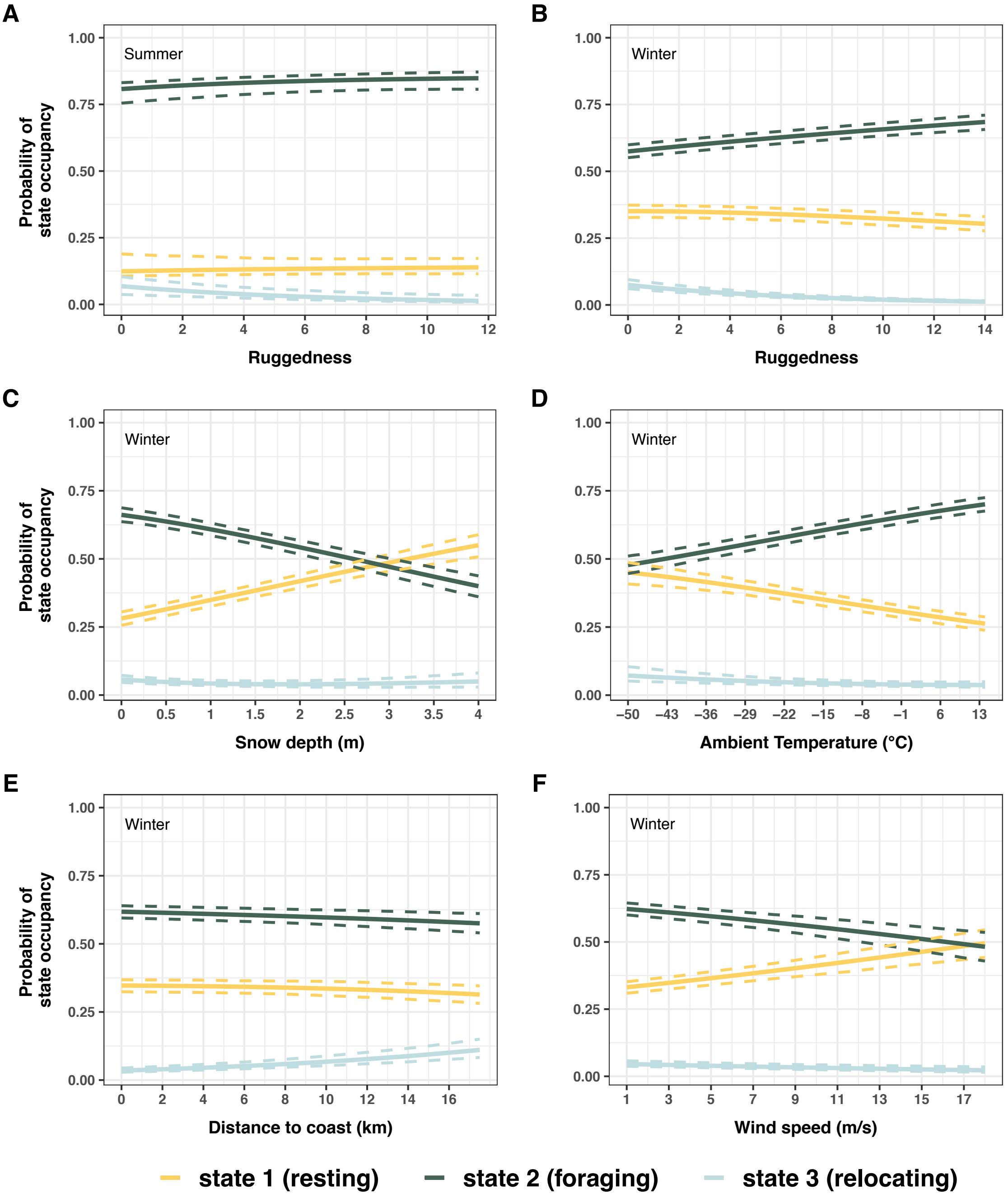

Figure S13: Probabilities (mean and 95% CI) of behavioural state occupancy as a function of the environmental covariates included in the BIC-selected bivariate HMMs for the (A) summer and (B-F) winter season. Probabilities were calculated for each covariate and state by fixing the values of the remaining continuous environmental covariates at their respective seasonal mean. Continuous temporal covariates were set to Julian Day 213 (i.e. August 1st) and 91 (i.e. April 1st) for summer and winter, respectively, and to12 o'clock for time of day. Landcover type was set to **dense vegetation**, and the remaining categorical covariates to their corresponding reference categories (i.e. daylight, and winter 2013-2014 for winter model). Monte Carlo simulation from the estimator's approximate multivariate normal distribution was used to obtain pointwise 95% CIs.

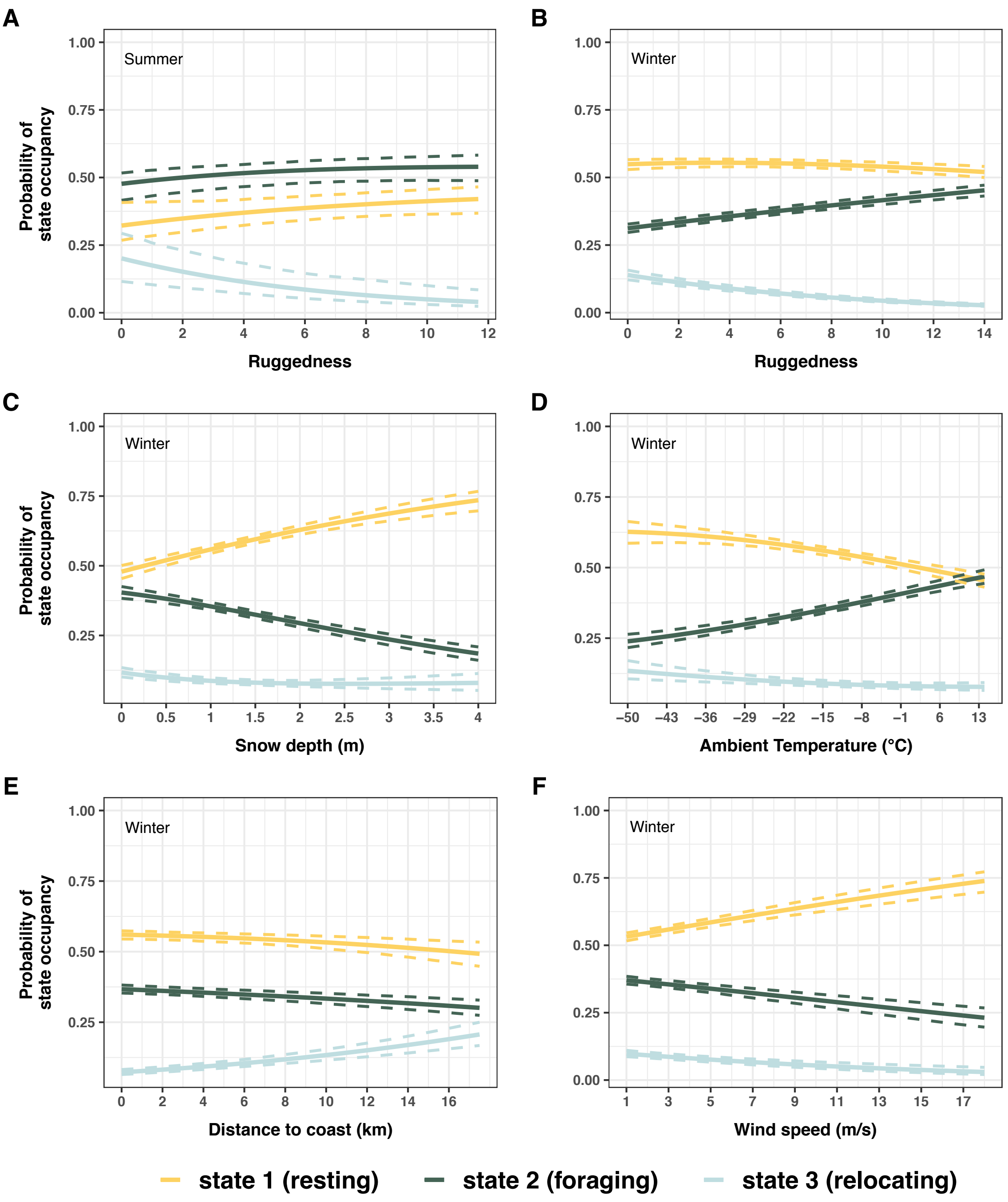

Figure S14: Probabilities (mean and 95% CI) of behavioural state occupancy as a function of the environmental covariates included in the BIC-selected bivariate HMMs for the (A) summer and (B-F) winter season. Probabilities were calculated for each covariate and state by fixing the values of the remaining continuous environmental covariates at their respective seasonal mean. Continuous temporal covariates were set to **Julian Day 243** (i.e. **August 31st**) and 91 (i.e. April 1st) for summer and winter, respectively, and to **0 o'clock** for time of day. Light was set to **darkness**, and the remaining categorical covariates to their corresponding reference categories (i.e. bare ground, and winter 2013-2014 for winter model). Monte Carlo simulation from the estimator's approximate multivariate normal distribution was used to obtain pointwise 95% CIs.

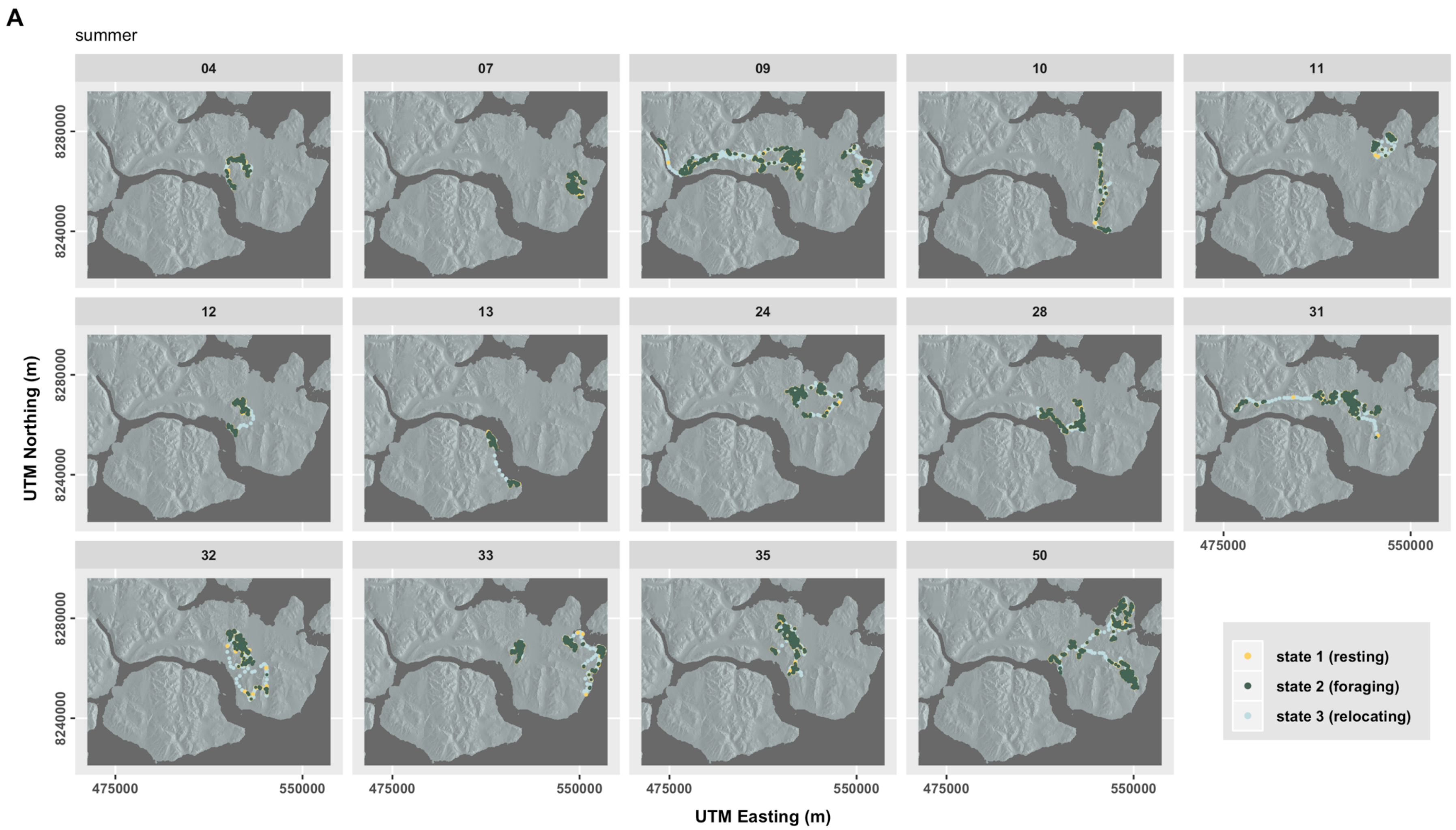
A
summer
04
07
09
10
11
12
13
24
28
31
32
33
35
50
UTM Northing (m)
8280000
8240000
UTM Easting (m)
475000
550000
state 1 (resting)
state 2 (foraging)
state 3 (relocating)

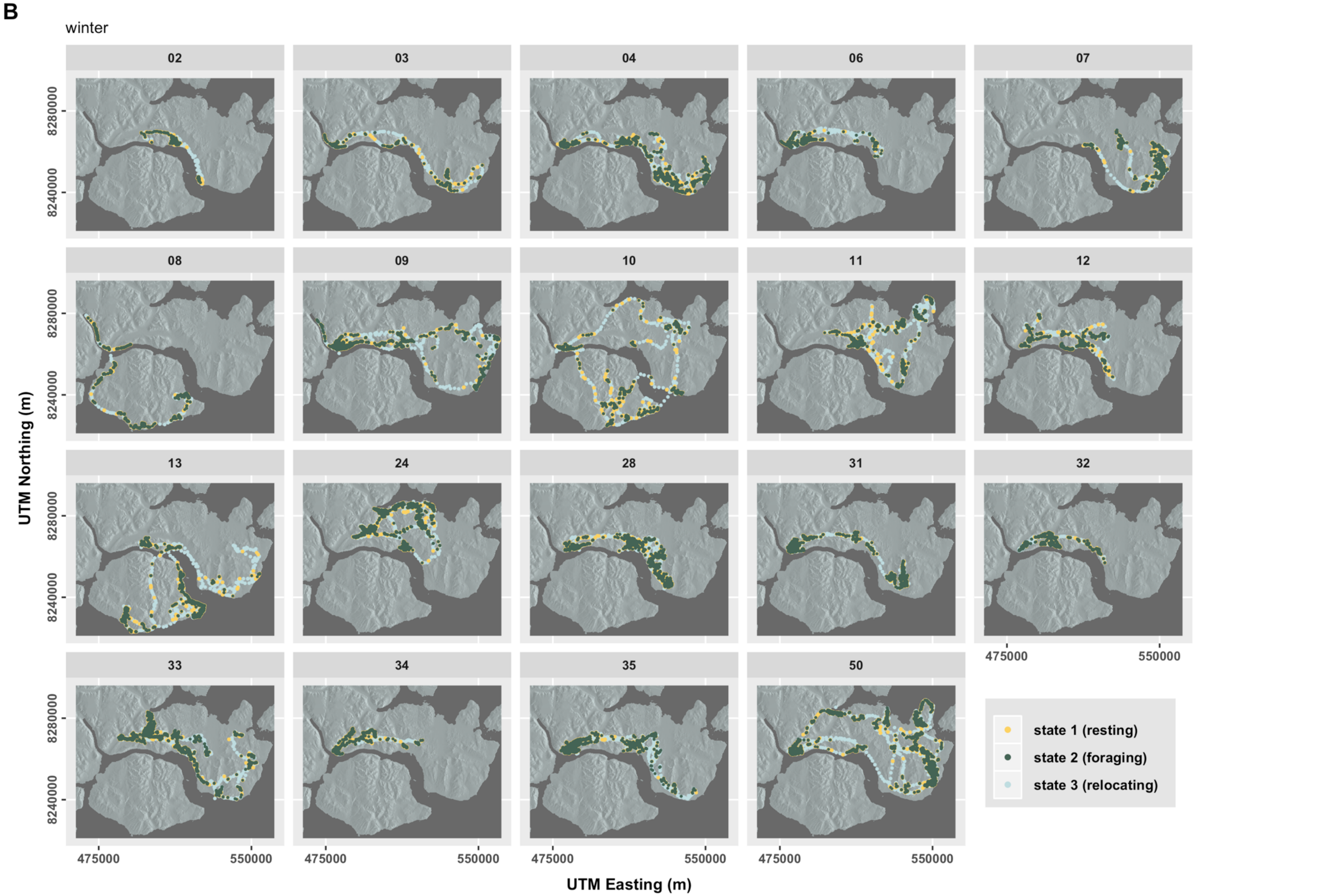

Figure S15: Maps showing muskox locations state-decoded based on the Viterbi algorithm during the (A) snow-free summer und (B) snow-covered winter season, by animal ID. Note that animals may have been tracked for more than one winter/summer season.

Table S1: Number of observations, observation days, number of bursts, tracks and animals per season included in analyses of muskox movement behaviour.

**season**	**n observations**	**observation days**	**n bursts**	**n tracks**	**n animals**
summer 2014	10368	432	10	10	10
summer 2015	4656	194	4	4	4
summer 2016	10120	422	6	6	6
summer 2017	3021	126	3	3	3
winter 2013/14	87074	3628	14	14	14
winter 2014/15	59459	2477	10	10	10
winter 2015/16	44928	1872	20	8	8
winter 2016/17	22752	948	3	3	3
total	242378	10099	70	19	19

Table S2: Coefficients (beta and standard error) of the multinomial logistic regression summer model that were used to predict the probability of muskox state occupancy as a function of the BIC-selected covariates (Figure 4 in main manuscript).

	**state 1 » 2**		**state 1 » 3**		**state 2 » 1**		**state 2 » 3**		**state 3 » 1**		**state 3 » 2**	
**parameter**	**beta**	**se**	**beta**	**se**	**beta**	**se**	**beta**	**se**	**beta**	**se**	**beta**	**se**
intercept	-0.48	0.72	-3.93	1.91	-0.86	0.41	-3.98	0.82	-9.91	2.54	-1.84	1.15
light*	0.9	0.11	1.39	0.81	-0.61	0.09	-0.03	0.23	1.13	1.77	-0.95	0.28
landcover type*												
sparse vegetation	0.11	0.1	-1.25	0.36	-0.33	0.06	-0.86	0.13	-1.26	0.27	1.11	0.18
dense vegetation	-0.12	0.15	-2.44	1.45	-0.49	0.09	-1.29	0.23	-6.29	10.51	2.6	0.34
ruggedness	-0.04	0.04	-0.1	0.14	0	0.03	-0.42	0.08	0.06	0.1	0.33	0.09
sin(Julian day)	0.46	0.51	0.48	1.01	-0.43	0.26	-1.17	0.49	-3.38	0.93	-1.32	0.71
cos(Julian day)	-1.36	0.59	-2.69	1.73	0.21	0.36	-1.62	0.73	-6.79	1.65	-0.52	1.01

* Reference category is daylight [light] and bare ground [landcover type]

Table S3: Coefficients (beta and standard error) of the multinomial logistic regression winter model that were used to predict the probability of muskox state occupancy as a function of the BIC-selected covariates (Figure 4 in main manuscript).

	**state 1 » 2**		**state 1 » 3**		**state 2 » 1**		**state 2 » 3**		**state 3 » 1**		**state 3 » 2**	
**parameter**	**beta**	**se**	**beta**	**se**	**beta**	**se**	**beta**	**se**	**beta**	**se**	**beta**	**se**
intercept	-1.1	0.03	-1.43	0.07	-1.34	0.03	-3.05	0.09	-1.93	0.08	-1.6	0.07
sin(Julian day)	-0.19	0.02	-0.43	0.06	-0.01	0.02	0.0	0.0	0.23	0.07	0.17	0.06
cos(Julian day)	-0.5	0.03	-1.46	0.09	0.1	0.03	-0.36	0.09	0.03	0.1	-0.45	0.09
sin(time of day)	-0.06	0.01	-0.01	0.04	-0.05	0.01	0.07	0.03	-0.13	0.05	-0.26	0.04
cos(time of day)	-0.28	0.02	-1.3	0.06	0.27	0.02	0.01	0.04	0.36	0.05	0.02	0.04
landcover type*												
sparse vegetation	0.19	0.02	-1.06	0.07	0.3	0.02	-0.83	0.06	0.12	0.07	0.93	0.06
dense vegetation	0.08	0.05	-1.48	0.17	0.38	0.05	-1.51	0.14	-0.19	0.32	1.56	0.14
ruggedness	0.07	0.01	-0.4	0.04	-0.07	0.01	-0.27	0.03	-0.04	0.04	0.23	0.03
snow depth	-0.13	0.02	-0.02	0.05	0.12	0.02	-0.43	0.03	0.02	0.05	-0.09	0.04
year*												
winter 2014/15	0.22	0.03	-0.4	0.07	-0.11	0.03	0.52	0.07	0.17	0.08	0.07	0.07
winter 2015/16	0.21	0.03	-0.96	0.1	0.01	0.03	0.49	0.07	0.3	0.09	0.25	0.07
winter 2016/17	0.4	0.04	-0.76	0.11	0.09	0.04	0.31	0.08	0.17	0.12	0.28	0.09
light*	0.36	0.04	-0.85	0.13	-0.13	0.04	0.82	0.12	-0.35	0.1	-0.17	0.09
temperature	0.2	0.02	-0.19	0.05	-0.08	0.02	0.04	0.05	0.01	0.06	-0.06	0.05
distance to sea	-0.03	0.01	0.25	0.03	0	0.01	0.08	0.03	0.01	0.03	-0.09	0.03
wind speed	-0.08	0.01	-0.11	0.03	0.01	0.01	-0.06	0.03	0.1	0.03	0.01	0.03

* Reference category is bare ground [landcover type], winter 2013/14 [year] and daylight [light]
